# Feature-PLPD-Aided Visual–Inertial Odometry for Low-Cost Embedded Systems

**DOI:** 10.3390/s26185940

**Published:** 2026-09-19

**Authors:** Ayoub Mamri, Abdelhafid El Hadri, Abdelaziz Benallegue, Khalil Hachicha

**Affiliations:** 1 LISV, UVSQ, Université Paris-Saclay, 78124 Vélizy-Villacoublay, France; 2 LIP6, CNRS, Sorbonne Universite, 75005 Paris, France

**Keywords:** visual–inertial odometry, motion estimation, heterogeneous architectures, hardware–software co-design, real-time embedded systems

## Abstract

Visual–Inertial Odometry (VIO) has become a key technology for motion estimation in robotics and autonomous systems. However, deploying accurate VIO pipelines on embedded platforms remains challenging due to the trade-off between estimation accuracy, real-time performance, and energy consumption. This paper presents a hardware-aware Feature-PLPD VIO framework that integrates a point-and-line visual front-end with a loosely coupled Error-State Extended Kalman Filter (ESEKF) back-end. The proposed approach follows Algorithm Architecture Adequacy (A3) principles to preserve estimation robustness while limiting computational and memory requirements. To investigate its scalability across the considered resource constraints, two end-to-end embedded implementations are developed: a performance-oriented stereo VIO system on a GPU-based platform using GPU-aware software design, and a frugality-oriented low-cost RGB-D-assisted monocular VIO system on an FPGA-based architecture using hardware–software co-design with depth scale correction. The stereo GPU-based implementation is evaluated offline in both outdoor and indoor environments and is additionally validated through real-time on-the-fly deployment on a Scout Mini robot, whereas the FPGA-based implementation is evaluated offline using the indoor VICON dataset. Experimental results demonstrate meter-level trajectory accuracy and real-time performance under strict resource constraints. Averaged over four KITTI sequences, the proposed ESEKF-based fusion reduces the translation and rotation ATE by approximately 25% and 18%, respectively, compared with the corresponding VO-only configuration, while a 10% reduction in translation ATE is achieved in the indoor VICON environment, resulting in a normalized ATE of 5.09% over the 23.77 m trajectory. Both embedded implementations sustain around 20 fps, and the architectural evaluation highlights complementary accuracy–runtime–energy trade-offs between the GPU- and FPGA-based solutions. These results demonstrate the feasibility of scaling the proposed embedded VIO framework toward resource- and energy-constrained robotic applications under the investigated experimental and hardware configurations.

## 1. Introduction

Visual Odometry (VO) [1] is a key component of modern perception systems, enabling motion estimation from image sequences and supporting higher-level tasks such as Simultaneous Localization and Mapping (SLAM) [2] and 3D reconstruction [3,4]. VO has become central to many applications including autonomous driving [5], augmented reality [6], and biomedical navigation [7,8]. Over the past decade, significant progress has been achieved through feature-based and optimization-based approaches such as ORB-SLAM3 [9], OKVIS [10], and VINS-Mono [6]. While these systems achieve impressive localization accuracy, their computational complexity often limits direct deployment on embedded platforms operating under strict power and resource constraints.

In practical robotic applications, embedded VO systems must satisfy three competing requirements: estimation accuracy, real-time capability, and energy efficiency. This trade-off becomes particularly critical for miniaturized and resource-constrained systems, such as micro-UAVs, micro-robots, and capsule endoscopes, where computational resources, memory, and energy budgets are severely limited. Improving accuracy typically increases computational and memory demands, whereas reducing algorithmic complexity to meet runtime and energy constraints may degrade robustness. Addressing this trade-off therefore requires a frugal system-level design that jointly considers algorithmic complexity, sensor fusion, and hardware architecture from the early stages of development.

The first dimension of this trade-off concerns estimation accuracy. In feature-based pipelines, estimation errors often originate in the image pre-processing stage, where inaccuracies in feature detection and tracking propagate through the pipeline and accumulate as drift. To improve robustness in structured and low-texture environments, several works have explored combining complementary geometric primitives such as point and line features [11]. Building upon this idea, we previously introduced a hardware-aware Feature-PLPD front-end designed to balance robustness and computational cost through Algorithm Architecture Adequacy (A3) [12]. Nevertheless, purely visual odometry remains sensitive to depth uncertainty, scale ambiguity, and sensor noise. Visual–Inertial Odometry (VIO) mitigates these limitations by integrating inertial measurements. Among available fusion strategies, tightly coupled approaches jointly optimize visual and inertial states [6,10], but at high computational cost. In contrast, filtering-based solutions such as the Error-State Extended Kalman Filter (ESEKF) [13] estimate error states without jointly optimizing full landmark maps, offering a computationally efficient alternative for resource-constrained embedded deployment.

The second and third dimensions of the trade-off concern real-time performance and energy efficiency. While recent visual SLAM and VIO systems have improved localization accuracy, their deployment on embedded robotic platforms remains challenging due to the computational demands of visual front-end processing and optimization pipelines [14,15]. To address these constraints, several works have explored hardware acceleration on heterogeneous architectures, including GPU-based implementations for feature extraction and tracking and FPGA-based pipelines for visual perception tasks [16,17,18,19,20]. However, most existing solutions focus on accelerating individual algorithmic blocks rather than designing the complete motion-estimation pipeline under embedded constraints. Progress toward low-cost embedded localization therefore requires explicit algorithm architecture co-design, where computational tasks, sensor fusion, and hardware mapping are jointly structured to exploit parallelism and heterogeneous accelerators while preserving estimation robustness.

The present work builds upon a systematic application of the A3 methodology through three successive developments toward complete embedded visual–inertial motion estimation. The original Feature-PLPD framework introduced a hardware-aware point–line visual front-end for embedded VO [21]. A subsequent work enhanced the tracking of line-segment points by exploiting their geometric and gradient properties to improve feature persistence [22]. In parallel, the portability of the computationally intensive visual front-end was investigated through an ARM–FPGA hardware–software co-design [23]. These developments addressed complementary aspects of embedded visual motion estimation, but remained limited to visual odometry or individual processing components. The present work integrates and extends these building blocks toward complete embedded VIO systems by introducing inertial fusion and evaluating the resulting pipelines jointly in terms of accuracy, runtime performance, and energy consumption. Table 1 summarizes the scope of these previous contributions and highlights the extensions introduced in the present work.

Following this frugal system-level design philosophy, two complementary end-to-end configurations are developed to investigate the scalability of the framework across different embedded constraints. The first is a high-performance stereo VIO system deployed on a GPU-based heterogeneous architecture, while the second moves toward lower-cost and more resource-constrained deployment through an RGB-D-assisted monocular VIO system implemented on an FPGA-based heterogeneous architecture with depth scale correction. Both systems integrate the Feature-PLPD front-end with a resource-aware ESEKF-based loosely coupled fusion strategy. Unlike conventional VIO systems relying on high-frequency inertial propagation or pre-integration, the ESEKF prediction is performed at the camera rate to limit computational and memory overhead. This deliberate design choice prioritizes deployment feasibility and portability across platforms with different resource and energy budgets.

The proposed systems are evaluated through hardware execution and dataset-based experiments, while the stereo GPU implementation is additionally assessed through real-time on-the-fly deployment on a robotic platform. The study progressively analyzes the transition from VO-only to complete VIO estimation and evaluates the resulting implementations in terms of trajectory accuracy, throughput, and energy consumption. Rather than targeting accuracy alone, the objective is to determine how hardware-aware visual processing, resource-aware sensor fusion, and heterogeneous acceleration can jointly pave the way toward end-to-end low-cost embedded motion estimation.

In summary, the main contributions of this work are as follows:

- The integration and extension of our previous hardware-aware visual processing developments into complete Feature-PLPD VIO pipelines, addressing the absence of inertial fusion and full-system deployment in the earlier studies.
- A resource-aware ESEKF-based loosely coupled fusion strategy, in which inertial prediction is performed at the camera rate to improve VO robustness while limiting the computational and memory overhead of high-frequency IMU processing.
- Two complementary end-to-end embedded implementations targeting different resource constraints: a high-performance GPU-based stereo VIO and a low-cost FPGA-based RGB-D-assisted monocular VIO with depth scale correction.
- A progressive experimental analysis from visual-only odometry and previously developed processing blocks to complete VIO implementations, together with dataset-based evaluation and on-the-fly robotic deployment of the stereo system.
- A joint quantitative evaluation of trajectory accuracy, throughput, and energy consumption to characterize the trade-offs involved in scaling the framework toward low-cost embedded motion estimation.

## 2. Related Work

### 2.1. Visual Odometry and Feature-Based Front-Ends

VO has long been a fundamental component of visual SLAM and motion estimation systems, starting from the geometric formulation introduced by Nistér [24]. Modern feature-based pipelines such as ORB-SLAM2 [25] and ORB-SLAM3 [9] rely on keypoint detection and descriptor matching to estimate camera motion with high accuracy. In contrast, direct approaches such as Direct Sparse Odometry (DSO) [26] estimate motion by minimizing photometric error directly on image intensities without explicit feature extraction. While these approaches achieve strong performance in well-textured scenes, their robustness can deteriorate in environments characterized by low texture, repetitive patterns, or strong geometric structures.

To address this limitation, several works have explored integrating line-segment features alongside point features. PL-SLAM [11] demonstrated that combining ORB keypoints with LSD-detected line segments improves robustness in structured environments. Similarly, PL-VIO [27] incorporates FAST corners together with LSD line detection within a tightly coupled visual–inertial estimation framework. PLD-VINS [28] further extends this concept by integrating Shi–Tomasi corner detection with EDLines for RGB-D visual–inertial SLAM, while the ray-to-ray formulation proposed in [29] improves the geometric consistency of line measurements in visual SLAM systems.

Although hybrid point–line representations improve robustness, the overall performance of VO/VIO pipelines strongly depends on the image pre-processing front-end, which determines the quality and spatial distribution of tracked features. To better analyze the impact of front-end design choices, we evaluate several representative configurations derived from state-of-the-art SLAM systems by isolating their visual odometry component. The comparison includes front-ends based on Shi–Tomasi features tracked with optical flow [30], ORB features combined with LSD line detection [11], FAST corners with LSD segments [27], and EDLines-based approaches used in PLD-VINS [28] and the ray-to-ray formulation [29]. For each configuration, the corresponding tracking strategy and VO-related parameters were adopted from the original method. Each VO configuration was implemented according to the corresponding original front-end formulation and evaluated under the same experimental conditions on the Jetson Xavier NX platform. For the detector-level performance comparison, CUDA implementations were used for the considered corner detectors (ORB, GFT, and FAST); these CUDA implementations concern only the standalone detector evaluation and were not used for the VO configurations. The results summarized in Table 2 highlight how different detection and tracking strategies affect motion estimation accuracy and computational performance.

Classical point-based pipelines offer efficient tracking but remain sensitive to visual degradation and structural ambiguities. Hybrid point–line approaches improve robustness in structured environments but often increase computational complexity due to the additional geometric primitives involved. In this context, the Feature-PLPD front-end recently introduced in [21] provides a favorable balance between feature quality and computational efficiency by jointly detecting corner features and line-based points. This design improves feature distribution and tracking robustness while maintaining real-time performance, making it particularly suitable for embedded visual odometry systems.

### 2.2. Visual–Inertial Odometry: Tightly and Loosely Coupled Approaches

The integration of inertial measurements further improves the robustness of motion estimation, particularly in scenarios where visual information becomes unreliable due to fast motion, motion blur, or poor illumination. Visual–Inertial Odometry (VIO) methods generally integrate visual and inertial data using either loosely coupled or tightly coupled fusion strategies.

In loosely coupled approaches, visual odometry and inertial measurements are estimated independently and fused at a higher level through probabilistic filtering frameworks such as the Extended Kalman Filter [31,32,33]. This strategy allows the visual and inertial pipelines to operate separately while compensating for visual drift through inertial measurements. As a result, loosely coupled systems typically require lower computational resources and are well suited for real-time implementations on embedded platforms.

Tightly coupled approaches, on the other hand, integrate visual observations and inertial measurements directly within a unified optimization or filtering framework. Systems such as OKVIS [10], ROVIO [34], and VINS-Mono [6] jointly estimate the system state by incorporating both visual and inertial constraints, leading to improved estimation accuracy and consistency. However, this tighter integration increases computational complexity and memory requirements, since both visual features and inertial measurements must be processed simultaneously during state estimation.

Table 3 further positions the proposed framework with respect to representative VIO systems and highlights the differences in their estimation strategies and experimental conditions. While most of these approaches primarily target robust state estimation through tightly coupled fusion, the proposed framework targets resource-aware embedded deployment by jointly considering trajectory accuracy, runtime performance, and energy consumption. For this purpose, we adopt a loosely coupled fusion approach based on an ESEKF, providing a lightweight alternative compatible with the computational and memory constraints of heterogeneous embedded platforms. A direct quantitative comparison at the complete VIO-system level is not considered here because the reported methods rely on different datasets, sensor configurations, inertial processing strategies, evaluation protocols, and computing platforms. Such heterogeneous conditions would not provide a consistent basis for numerical performance ranking. Instead, a controlled quantitative comparison is provided at the visual front-end level in Table 2, where the considered configurations are evaluated under the same experimental conditions. At the complete system level, the experimental evaluation therefore focuses on the accuracy–runtime–energy trade-off of the proposed VIO implementations under their targeted embedded resource constraints.

### 2.3. Embedded Implementations of VO and VIO

The deployment of VO/VIO systems on embedded platforms has attracted increasing attention due to the growing demand for onboard perception in mobile robots and autonomous systems. However, the image pre-processing front-end of VO pipelines remains computationally demanding, requiring careful optimization to satisfy real-time and resource constraints.

Several works have explored GPU-aware implementations to accelerate computationally intensive blocks of VO and SLAM pipelines. Ondruška et al. [16] proposed a dense tracking system exploiting GPU acceleration for stereo depth computation and volumetric model updates. Lin et al. [17] developed an indirect visual odometry system optimized for the NVIDIA Jetson TX2 platform with GPU-parallelized feature extraction and matching. Similarly, Nagy et al. [18] accelerated FAST detection and KLT optical flow tracking using CUDA kernels to improve the runtime of visual–inertial odometry pipelines. Hybrid CPU–GPU implementations have also been explored; for example, Nguyen et al. [35,36] proposed a bio-inspired feature extraction method combining FAST detection with a Hessian score and an amended FREAK descriptor [37], where feature extraction runs on the CPU while feature matching is accelerated on the GPU. Other works focused on porting existing SLAM frameworks to GPUs, such as the GPU implementations of ORB-SLAM2 proposed in [38,39].

Alternatively, FPGA-based accelerators have been investigated to improve energy efficiency and deterministic latency in robotic perception systems. Wan et al. [40] provide a comprehensive overview of FPGA-based robotic computing architectures, highlighting their potential for accelerating perception pipelines under strict resource constraints. Several studies have implemented hardware pipelines for visual front-end algorithms, including ORB feature extraction [41] and LSD-based line detection [19]. Other works explored FPGA implementations of edge-based motion estimation and feature tracking [20,42]. While these approaches achieve high throughput and energy efficiency, they often require rewriting algorithmic components in hardware description languages such as VHDL or Verilog, which increases development complexity and reduces portability.

To address these limitations, hardware–software co-design approaches have been proposed to combine the flexibility of embedded processors with the acceleration capabilities of heterogeneous architectures. Schulz et al. [43] implemented a Harris corner detector on a Zynq SoC using a pipelined FPGA architecture coupled with ARM-based pre-processing. Nguyen et al. [44] demonstrated a heterogeneous ARM–FPGA implementation of the HOOFR feature extractor using OpenCL, while Shi et al. [45] introduced the HERO heterogeneous computing platform for accelerating robotic perception pipelines. Abouzahir et al. [46] further proposed an end-to-end FastSLAM2.0 implementation on a CPU–FPGA architecture and highlighted the benefits of heterogeneous acceleration for robotic perception systems. More recently, Eisoldt et al. [47] demonstrated an energy-efficient LiDAR-based SLAM system implemented on a heterogeneous CPU–FPGA platform, evaluating runtime, power consumption, and energy per scan in embedded robotic scenarios.

Despite these advances, few works jointly address robust point–line visual processing, efficient visual–inertial fusion, and heterogeneous embedded deployment while simultaneously evaluating accuracy, runtime performance, and energy consumption. In contrast, the proposed framework integrates these aspects within a unified algorithm architecture co-design approach validated on heterogeneous embedded platforms.

## 3. Method

In this section, an overview of the Feature-PLPD tracking front-end is presented in Section 3.1. Then, the proposed visual–inertial fusion strategy is introduced in Section 3.2. Finally, Section 3.3 and Section 3.4 describe the embedded implementations of the proposed system on GPU-based and FPGA-based heterogeneous architectures, respectively.

### 3.1. Feature-PLPD Tracking Overview

**Figure 1:**
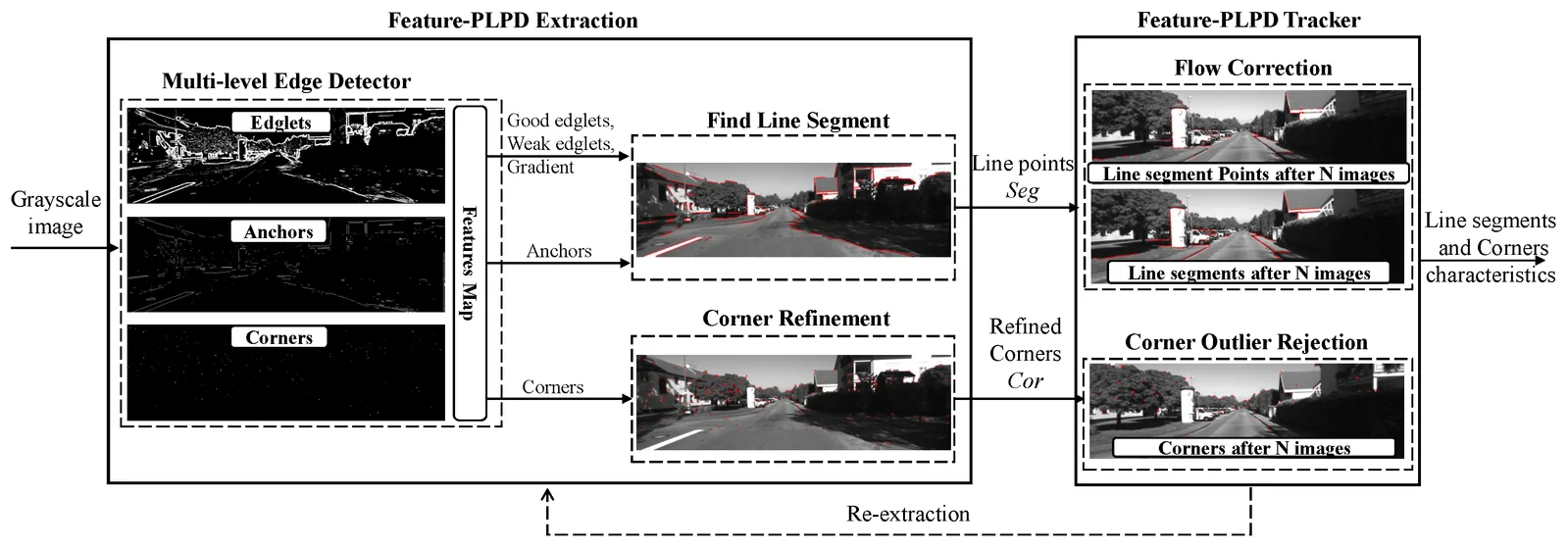
Image pre-processing front-end flowchart represented by our Feature-PLPD Tracking.

Feature-PLPD Tracking front-end, as illustrated in Figure 1, allows extracting a set of features (Points/Line points) from a grayscale image of the scene and afterward ensures their tracking in the subsequent images until the number of features becomes insufficient, which subsequently prompts a re-extraction in the next inserted image and then repeats the process until the end of the image sequence.

The process consists of two main threads:

- **The Feature-PLPD Extraction thread** begins by using our Feature-Point (corners) and Line Point (segments) Detector [21] for initialization, ensuring enough long-line segments and corners. If features are insufficient, re-extraction occurs. The thread employs the Multi-level Edge Detector (MED) to create a pre-refined feature map of potential corners and edglets, then identifies line segments (FLS) by using a refined random anchor and orientation to grow the segment. Simultaneously, a corner refinement (CR) process identifies the true corners.
- **The Feature-PLPD Tracker thread** utilizes the sparse KLT optical flow algorithm (OF) [48] to estimate feature locations between frames using the Taylor equation. Due to its sensitivity to abrupt movement and noise, which can lead to point loss, we implemented a flow correction method (FC) [22] based on a line-fitting model that stabilizes line points and removes outliers through anchor and line-segment properties. Unlike line segments, corners lack a defined model, so we use a corner outlier rejection mechanism (COR) based on circular matching [30] or geometric modeling via RANSAC [49], depending on whether the camera is monocular or stereo, as detailed in Section 3.3 and Section 3.4.

### 3.2. Proposed Feature-PLPD VIO Back-End

**Figure 2:**
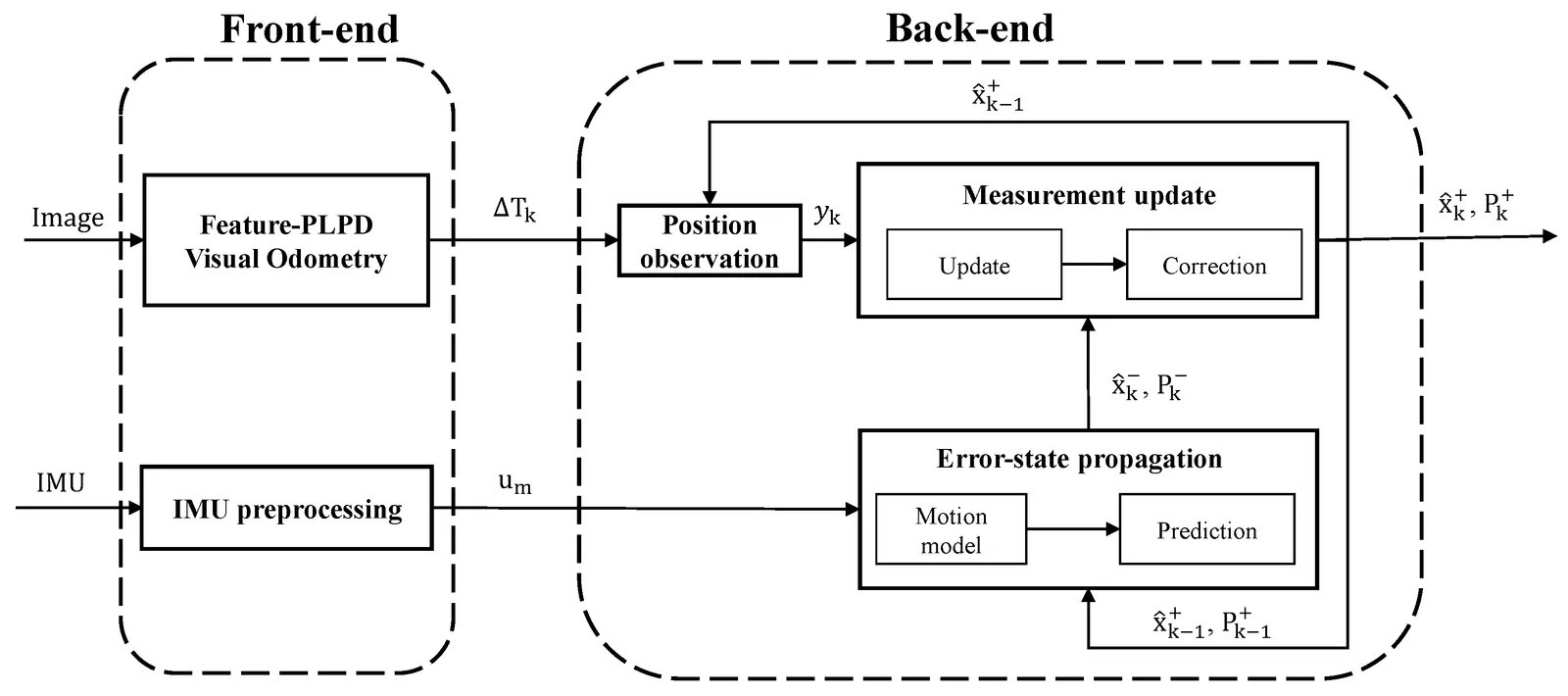
Proposed Feature-PLPD VIO flowchart.

While the Feature-PLPD image pre-processing front-end significantly enhances feature extraction and robustness, it does not fully eliminate drift in motion estimation, particularly in long-term trajectories. To ensure accurate localization, an effective state estimation framework is required to correct accumulated errors in VIO. The objective is to correct the error states of the VO estimates between two successive frames by integrating accelerometer and gyroscope measurements within the kinematic model as shown in Figure 2.

In this section, we present the mathematical formulation of ESEKF, detailing its state representation, propagation, and measurement update within a loosely coupled VIO framework.

In the proposed resource-aware implementation, the ESEKF prediction is performed at the camera rate using temporally aligned inertial measurements, rather than propagating the filter at the full IMU sampling rate. The proposed fusion is intentionally vision-centered: visual odometry remains the primary motion-estimation source, while inertial measurements provide complementary motion information, particularly under geometrically unfavorable conditions such as weakly observable parallax or near-pure rotational motion. At the camera rate, the inertial input used in the prediction represents the motion over a larger propagation interval than in conventional high-rate inertial propagation. This approximation is suitable when the inertial dynamics remain sufficiently smooth between consecutive image timestamps, but it does not capture intermediate variations of acceleration and angular velocity. Consequently, the resulting prediction may be less accurate under rapid or highly dynamic motion. In such conditions, the unmodeled intermediate inertial variations may also make the propagated covariance less representative of the actual motion uncertainty over the camera interval. High-frequency IMU propagation or pre-integration remains applicable when supported by the computational and memory resources of the target platform and can better account for these intermediate inertial dynamics. However, the present work targets a frugal VIO design intended to remain transferable to miniaturized and resource-constrained embedded systems. The adopted strategy is therefore not motivated solely by the execution cost of the ESEKF, but by a system-level design objective in which the inertial-processing complexity is deliberately kept minimal while retaining sufficient inertial information to complement the visual estimate.

#### 3.2.1. ESEKF Kinematic Model in Discrete Time

The nominal state vector is defined as

(1) $$x={\left(\begin{array}{c} p^{T}\;v^{T}\;q^{T}\;a_{b}^{T}\;w_{b}^{T} \end{array}\right)}^{T}$$

where

- $p\in R^{3}$ is the position of the mobile platform in the global frame;
- $v\in R^{3}$ is the velocity in the global frame;
- $q\in R^{4}$ is the orientation quaternion from the body frame to the global frame;
- $a_{b}\in R^{3}$ is the accelerometer bias;
- $\omega_{b}\in R^{3}$ is the gyroscope bias.

and the different equations of the nominal state kinematics can be written as

(2) $$\left\{\begin{array}{cc} p_{k} & \leftarrow p_{k-1}+v_{k-1}\Delta t+\frac{1}{2}\left(R\left\{q_{k-1}\right\}\left(a_{m,k-1}-a_{b,k-1}\right)+g\right)\Delta t^{2}\\ v_{k} & \leftarrow v_{k-1}+\left(R\left\{q_{k-1}\right\}\left(a_{m,k-1}-a_{b,k-1}\right)+g\right)\Delta t\\ q_{k} & \leftarrow q_{k-1}⊗\exp\left(\frac{1}{2}\left(\omega_{m,k-1}-\omega_{b,k-1}\right)\Delta t\right)\\ a_{b_{k}} & \leftarrow a_{b,k-1}\\ \omega_{b_{k}} & \leftarrow \omega_{b,k-1} \end{array}\right.$$

with $u_{m}=\left[a_{m,k-1},\omega_{m,k-1}\right]$ is the IMU measurement (i.e., accelerometer and gyroscope). Instead of estimating the nominal state x directly, ESEKF tracks only small perturbations (errors) in the state, represented as the error-state vector, which is defined by the difference between the nominal state x and its estimate $\hat{x}$ as

(3) $$\delta x=x-\hat{x}={\left(\begin{array}{c} \delta p^{T}\;\delta v^{T}\;\delta \theta^{T}\;\delta a_{b}^{T}\;\delta w_{b}^{T} \end{array}\right)}^{T}$$

where δp and δv are the position and velocity errors, δθ represents the small-angle orientation perturbation, modeled using an axis-angle representation rather than quaternions for computational efficiency, and $\delta a_{b}$ and $\delta \omega_{b}$ are the accelerometer and gyroscope bias errors. The attitude error follows a right-multiplicative convention, such that $R\left(q\right)=R\left(\hat{q}\right)\mathrm{Exp}\left({\left[\delta \theta \right]}_{\times }\right)$, consistently with the error injection used during the correction step. The equation of the error-state kinematics can be written as

(4) $$\left\{\begin{array}{cc} \delta p_{k} & =\delta p_{k-1}+\delta v_{k-1}\Delta t\\ \delta v_{k} & =\delta v_{k-1}-\left(R_{k-1}S\left(a_{m,k-1}-a_{b,k-1}\right)\delta \theta_{k-1}+R_{k-1}\delta a_{b,k-1}\right)\Delta t+v_{i}\\ \delta \theta_{k} & =R^{T}\left\{\left(\omega_{m,k-1}-\omega_{b,k-1}\right)\Delta t\right\}\delta \theta_{k-1}-\delta \omega_{b,k-1}\Delta t+\theta_{i}\\ \delta a_{b,k} & =\delta a_{b,k-1}+a_{i}\\ \delta \omega_{b,k} & =\delta \omega_{b,k-1}+\omega_{i} \end{array}\right.$$

where $R_{k-1}≜R\left\{q_{k-1}\right\}$ denotes the rotation matrix from the body frame to the global frame, S(·) is the skew matrix operator, R{·}≜Exp(·) is the Rodrigues operator, and $v_{i},\theta_{i},a_{i},w_{i}\in R^{3}$ represent the discrete process-noise increments affecting velocity, orientation, accelerometer bias, and gyroscope bias, respectively, and are modeled as Gaussian noise. This state representation is written in a compact form as

(5) $$\delta x_{k}=F\delta x_{k-1}+Gn_{k-1}$$

with the model Jacobian

(6) $$F=\left[\begin{array}{ccccc} I_{3} & I_{3}\Delta t & 0_{3\times 3} & 0_{3\times 3} & 0_{3\times 3}\\ 0_{3\times 3} & I_{3} & -R_{k-1}S\left(a_{m,k-1}-a_{b,k-1}\right)\Delta t & -R_{k-1}\Delta t & 0_{3\times 3}\\ 0_{3\times 3} & 0_{3\times 3} & R^{T}\left\{\left(\omega_{m,k-1}-\omega_{b,k-1}\right)\Delta t\right\} & 0_{3\times 3} & -I_{3}\Delta t\\ 0_{3\times 3} & 0_{3\times 3} & 0_{3\times 3} & I_{3} & 0_{3\times 3}\\ 0_{3\times 3} & 0_{3\times 3} & 0_{3\times 3} & 0_{3\times 3} & I_{3} \end{array}\right]$$

and the gain matrix

(7) $$G=\left[\begin{array}{cccc} 0_{3\times 3} & 0_{3\times 3} & 0_{3\times 3} & 0_{3\times 3}\\ I_{3} & 0_{3\times 3} & 0_{3\times 3} & 0_{3\times 3}\\ 0_{3\times 3} & I_{3} & 0_{3\times 3} & 0_{3\times 3}\\ 0_{3\times 3} & 0_{3\times 3} & I_{3} & 0_{3\times 3}\\ 0_{3\times 3} & 0_{3\times 3} & 0_{3\times 3} & I_{3} \end{array}\right]$$

of the process noise $n_{k}={\left(v_{i}^{T}\theta_{i}^{T}a_{b_{i}}^{T}w_{b_{i}}^{T}\right)}^{T}$, which is supposed to be

(8) $$n_{k}∼N\left(0,Q_{k}\right)$$

with

(9) $$Q_{k}=\left[\begin{array}{cccc} \sigma_{\delta a}^{2}\Delta t^{2}I_{3} & 0_{3\times 3} & 0_{3\times 3} & 0_{3\times 3}\\ 0_{3\times 3} & \sigma_{\delta w}^{2}\Delta t^{2}I_{3} & 0_{3\times 3} & 0_{3\times 3}\\ 0_{3\times 3} & 0_{3\times 3} & \sigma_{\delta a_{b}}^{2}\Delta t^{2}I_{3} & 0_{3\times 3}\\ 0_{3\times 3} & 0_{3\times 3} & 0_{3\times 3} & \sigma_{\delta w_{b}}^{2}\Delta t^{2}I_{3} \end{array}\right]$$

where $\sigma_{\delta a}^{2}$, $\sigma_{\delta \omega }^{2}$, $\sigma_{\delta a_{b}}^{2}$, and $\sigma_{\delta \omega_{b}}^{2}$ denote the variances of the corresponding discrete noise terms associated with the accelerometer, gyroscope, accelerometer-bias, and gyroscope-bias processes, respectively. These quantities are sensor-dependent noise characteristics and are set according to the specifications of the IMU used in each experimental configuration. Their appropriate tuning also accounts for the expected platform dynamics and sampling conditions, since more dynamic motion may introduce larger propagation uncertainty than stationary or approximately constant-velocity motion. Since the corresponding noise increments are integrated over the sampling interval Δt, their contribution to the discrete process-noise covariance scales with $\Delta t^{2}$.

#### 3.2.2. ESEKF Algorithm

At system startup, the visual odometry initially operates independently while the ESEKF is initialized, including the estimation of the IMU bias terms. Once the filter initialization is completed, the visual pose estimates are incorporated into the correction step and the complete visual–inertial estimation is performed.

##### Prediction Step

- **Input**: Previous corrected estimate ${\hat{x}}_{k-1}^{+}=\left[{\hat{p}}_{k-1}{\hat{v}}_{k-1}{\hat{q}}_{k-1}{\hat{a}}_{b,k-1}{\hat{\omega }}_{b,k-1}\right]$, $P_{k-1}^{+}$; IMU measurement $u_{m}=\left[a_{m,k-1},\omega_{m,k-1}\right]$.
- **Output**: Predicted estimate ${\hat{x}}_{k}^{-}=\left[{\hat{p}}_{k}{\hat{v}}_{k}{\hat{q}}_{k}{\hat{a}}_{b,k}{\hat{\omega }}_{b,k}\right]$, $P_{k}^{-}$.

Hereafter, the superscripts − and + denote the predicted (prior) and corrected (posterior) estimates, respectively.

1. **Nominal State Propagation:**
$$\begin{array}{cc} {\hat{p}}_{k} & ={\hat{p}}_{k-1}+{\hat{v}}_{k-1}\Delta t+\frac{1}{2}\left({\hat{R}}_{k-1}\left(a_{m_{k-1}}-{\hat{a}}_{b_{k-1}}\right)+g\right)\Delta t^{2}\\ {\hat{v}}_{k} & ={\hat{v}}_{k-1}+\left({\hat{R}}_{k-1}\left(a_{m,k-1}-{\hat{a}}_{b,k-1}\right)+g\right)\Delta t\\ {\hat{q}}_{k} & ={\hat{q}}_{k-1}⊗\exp\left(\frac{1}{2}\left(\omega_{m,k-1}-{\hat{\omega }}_{b,k-1}\right)\Delta t\right)\\ {\hat{a}}_{b,k} & ={\hat{a}}_{b,k-1}\\ {\hat{\omega }}_{b,k} & ={\hat{\omega }}_{b,k-1} \end{array}$$
2. **Covariance Propagation:**
$$P_{k}^{-}=E\left[\delta x_{k}^{-}\delta x_{k}^{-⊤}\right]=FP_{k-1}^{+}F^{⊤}+GQ_{k-1}G^{⊤}$$

##### Correction Step (On Measurement)

- **Input**: Predicted estimate ${\hat{x}}_{k}^{-}=\left[{\hat{p}}_{k}^{-}{\hat{v}}_{k}^{-}{\hat{q}}_{k}^{-}{\hat{a}}_{b,k}^{-}{\hat{\omega }}_{b,k}^{-}\right]$, $P_{k}^{-}$; position measurement $y_{k}=\left[p_{m,k},v_{m,k},\theta_{m,k}\right]$.
- **Output**: Corrected estimate ${\hat{x}}_{k}^{+}=\left[{\hat{p}}_{k}^{+}{\hat{v}}_{k}^{+}{\hat{q}}_{k}^{+}{\hat{a}}_{b,k}^{+}{\hat{\omega }}_{b,k}^{+}\right]$, $P_{k}^{+}$.

Before proceeding with the correction steps, the measurement residual Jacobian matrix $H_{k}$ and measurement noise covariance matrix $R_{\nu_{k}}$ must be defined. Given the position measurement $y_{k}=h\left({\hat{x}}_{k}^{-}\right)+H_{k}\delta x_{k}+\nu_{k}$,

$$\begin{array}{cc} {\tilde{y}}_{k}=y_{k}⊖{\hat{y}}_{k}^{-} & =\left(\begin{array}{c} {\tilde{y}}_{p_{k}}=p_{m,k}-{\hat{p}}_{k}^{-}=\delta p_{k}+\nu_{p,k}\\ {\tilde{y}}_{v_{k}}=v_{m,k}-{\hat{v}}_{k}^{-}=\delta v_{k}+\nu_{v,k}\\ {\tilde{y}}_{\theta_{k}}=\frac{1}{2}vex\left(R_{m,k}^{T}{\hat{R}}_{k}^{-}-{\hat{R}}_{k}^{-T}R_{m,k}\right) \end{array}\right)\\ \; & =\left(\begin{array}{c} {\tilde{y}}_{p_{k}}=\delta p_{k}+\nu_{p,k}\\ {\tilde{y}}_{v_{k}}=\delta v_{k}+\nu_{v,k}\\ {\tilde{y}}_{\theta_{k}}=-\delta \theta_{L,k}+\nu_{\theta ,k} \end{array}\right) \end{array}$$

$$H_{k}=\left[\begin{array}{ccccc} I_{3} & 0 & 0 & 0 & 0\\ 0 & I_{3} & 0 & 0 & 0\\ 0 & 0 & -I_{3} & 0 & 0 \end{array}\right]$$

where vex(·) is an operator that extracts the elements of a skew matrix into their vectorial form. Note that the camera frame is considered as the body frame for the visual–inertial fusion and follows the convention *X*-right, *Y*-down, and *Z*-forward. The Feature-PLPD front-end estimates the relative camera motion $\Delta T_{k}$ from the previous camera frame to the current camera frame. Following the corrected-state feedback principle used in loosely coupled VO/INS fusion [50], this relative motion is composed with the previous ESEKF-corrected pose estimate, represented by the rigid-body transformation matrix ${\hat{T}}_{k-1}^{+}=T\left\{{\hat{x}}_{k-1}^{+}\right\}\in SE\left(3\right)$, reconstructed from the position and orientation components of the posterior corrected state ${\hat{x}}_{k-1}^{+}$, to construct the current visual pose measurement (as illustrated in Figure 2):

(10) $$\begin{array}{cc} y_{k}=\left[p_{m,k},v_{m,k},\theta_{m,k}\right]=T_{m,k}\equiv & {\hat{T}}_{k-1}^{+}\;\Delta T_{m,k}^{-1}\\ \equiv & {\hat{T}}_{k-1}^{+}{\left(\Delta T_{k}⊕\nu_{k}\right)}^{-1} \end{array}$$

where $p_{m,k}=t\left\{T_{m,k}\right\}$, $v_{m,k}=\frac{t\left\{T_{m,k}\right\}-t\left\{T_{m,k-1}\right\}}{\Delta time}$, and $\theta_{m,k}\equiv R\left\{T_{m,k}\right\}$ denote the position, velocity, and orientation components of the visual pose measurement, respectively.

It should be noted that, since the visual pose measurement in Equation (10) is reconstructed using the previous corrected ESEKF pose ${\hat{T}}_{k-1}^{+}$, the resulting visual pseudo-measurement is not strictly statistically independent of the filter state, as both share the uncertainty associated with ${\hat{T}}_{k-1}^{+}$. In the proposed loosely coupled implementation, this cross-correlation is neglected, and the conventional ESEKF measurement update is applied under the approximation $E[\delta x_{k}^{-}\nu_{k}^{T}]≃0$. Explicitly accounting for this cross-correlation would require an additional covariance propagation mechanism or an augmented-state formulation, thereby increasing the computational and memory requirements.

The measurement-noise vector $\nu_{k}={\left(\nu_{p_{k}}^{T}\nu_{v_{k}}^{T}\nu_{\theta_{k}}^{T}\right)}^{T}$, associated with the position, velocity, and orientation measurements, is modeled as Gaussian noise:

(11) $$\nu_{k}={\left(\nu_{p_{k}}^{T}\nu_{v_{k}}^{T}\nu_{\theta_{k}}^{T}\right)}^{T}∼N\left(0,R_{\nu_{k}}\right)$$

where $R_{\nu_{k}}$ denotes the measurement-noise covariance associated with the visual pose estimate. It is derived from the reprojection residuals of the retained feature correspondences by linearizing them with respect to a minimal 6-DoF pose perturbation. Denoting by $J_{k}$ the corresponding reprojection Jacobian and by $W_{k}$ the observation weighting matrix, the visual pose covariance is locally approximated from the inverse information matrix as

(12) $$R_{\nu_{k}}≃{\left(J_{k}^{T}W_{k}J_{k}\right)}^{-1}.$$

This formulation reflects the uncertainty associated with the geometry and reprojection residuals of the visual observations in both configurations, i.e., the stereo configuration described in Section 3.3 and the RGB-D-assisted monocular configuration described in Section 3.4. In the latter, the covariance is evaluated after applying the depth-based metric scale to the estimated translation.

Once $H_{k}$ and $R_{\nu_{k}}$ are defined, the correction step is applied as follows:

1. **Kalman Gain:**
  $$K_{k}=P_{k}^{-}H_{k}^{⊤}{\left(H_{k}P_{k}^{-}H_{k}^{⊤}+R_{\nu_{k}}\right)}^{-1}$$
2. **Error-State Correction:**
  $$\delta x_{k}=K_{k}{\tilde{y}}_{k}$$
3. **Inject Error into Nominal State:**
  $$\begin{array}{cc} {\hat{p}}_{k}^{+} & \leftarrow {\hat{p}}_{k}^{-}+\delta p_{k}\\ {\hat{v}}_{k}^{+} & \leftarrow {\hat{v}}_{k}^{-}+\delta v_{k}\\ {\hat{q}}_{k}^{+} & \leftarrow {\hat{q}}_{k}^{-}⊗\exp\left(\frac{1}{2}\left(\delta \theta_{k}\right)\right)\\ {\hat{a}}_{b,k}^{+} & \leftarrow {\hat{a}}_{b,k}^{-}+\delta a_{b,k}\\ {\hat{\omega }}_{b,k}^{+} & \leftarrow {\hat{\omega }}_{b,k}^{-}+\delta \omega_{b,k} \end{array}$$
4. **Covariance Update:**
  $$P_{k}^{+}=E\left[\delta x_{k}^{+}\delta x_{k}^{+⊤}\right]=\left(I-K_{k}H_{k}\right)P_{k}^{-}$$

The ESEKF efficiently corrects motion estimation errors by propagating error states and updating the pose using visual measurements. Unlike full-state filtering, it provides computational advantages crucial for real-time embedded platforms, making it an ideal choice for the proposed Feature-PLPD stereo and RGB-D-assisted monocular VIO systems.

The following sections will explore two embedded implementations of Feature-PLPD VIO with ESEKF, optimized for stereo VIO on a GPU-based heterogeneous platform and RGB-D-assisted monocular VIO on an FPGA-based system, demonstrating the effectiveness of this approach in real-world scenarios.

### 3.3. End-to-End Embedded Feature-PLPD Stereo VIO on GPU-Based Heterogeneous Architecture

**Figure 3:**
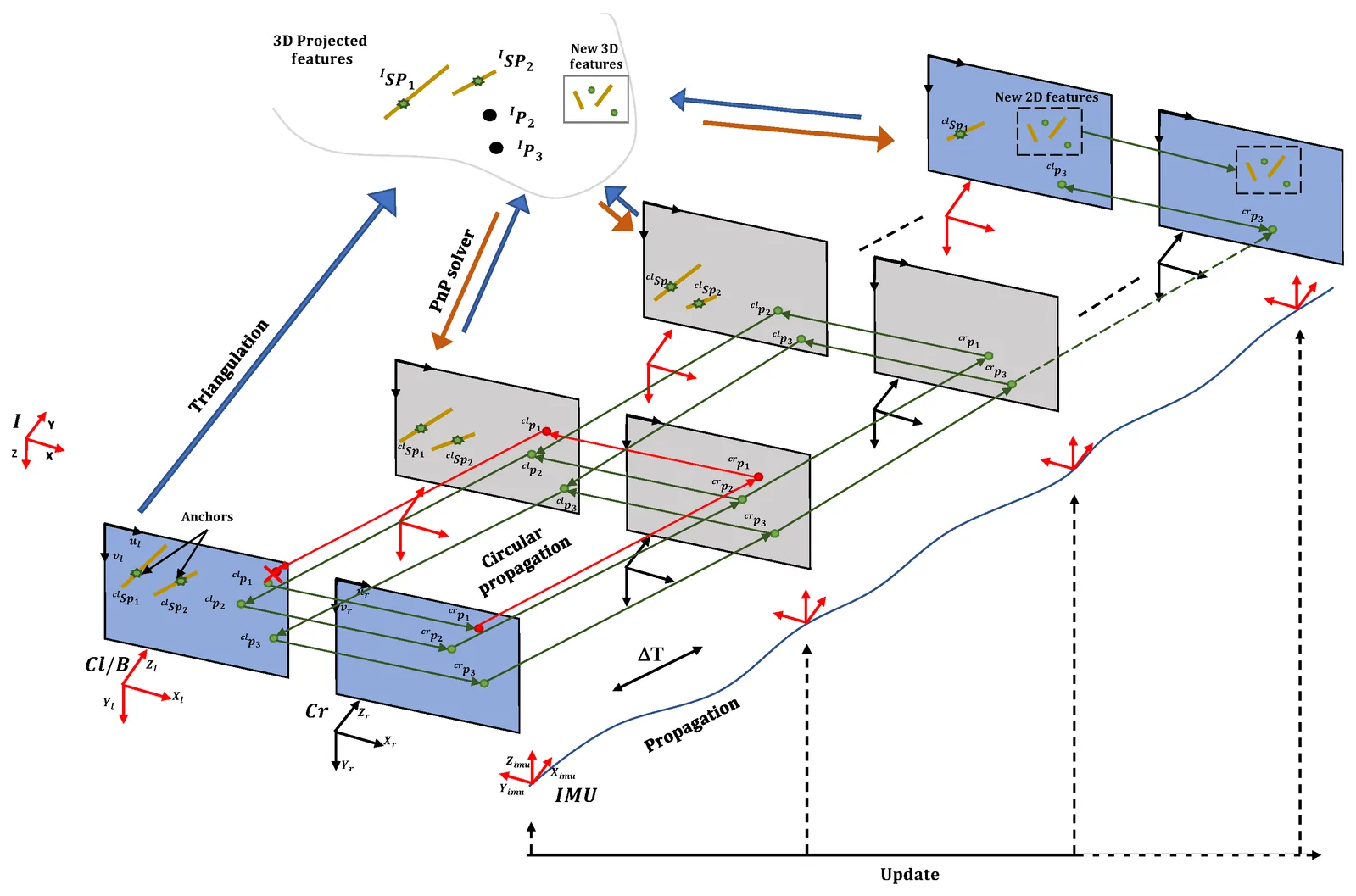
Feature-PLPD Stereo VIO process illustration, with blue frames indicating the feature extraction phase, while the gray frames denote the feature tracking phase. Green and red lines indicate the propagation of retained and rejected corners, respectively, while dashed lines indicate the continuity of the process across successive steps.

The system employs a stereo camera configuration that captures synchronized left and right image frames, in conjunction with an IMU that provides high-frequency measurements of linear acceleration and angular velocity. Our implementation incorporates a synchronization mechanism to ensure the alignment of timestamps between visual and inertial data, which is essential for accurate and rapid sensor fusion. As illustrated in Figure 3, we present the proposed Feature-PLPD image pre-processing process across multiple frames, designating the left frame as the body frame (*Cl*/*B*).

The initialization and proposal process is structured as follows:

1. **Extraction**: The process begins with the extraction of a set of features (corners $p_{i}^{cl}$ and line-segment points $Sp_{i}^{cl}$) from the previous left camera frame.
2. **Tracking**: The forward tracking of line-segment points is conducted using the proposed OF-based Flow correction method and ensures their stereo correspondence in the prior right camera. Simultaneously, corners are propagated in a circular manner across the four frames (previous left camera → previous right camera → current right camera → current left camera, and back to the previous left camera) to facilitate outlier rejection.
3. **Triangulation**: The aforementioned step results in feature correspondences between the previous left and right frames, which enables 3D projection through triangulation based on intrinsic camera parameters.
4. **PnP solver**: The feature tracking from Step 2, alongside the triangulation results from Step 3, provides the necessary input for the PnP solver to minimize the 3D-2D projection error within the current left camera. This involves comparing the current 2D features with their re-projected counterparts (3D to 2D). The PnP solver, supported by a complementary RANSAC method for outlier rejection, effectively resolves the optimization problem and estimates the relative pose between previous and current frames (ΔT). Then, the current position measurement $y_{k}$ can be computed by Equation (10).
5. **IMU propagation**: Concurrently, the IMU data is propagated through the adopted ESEKF-based observer in the back-end across the sequence frames to refine the pose estimation. This approach corrects accumulated drift by updating the error states related to position, orientation, and velocity, thereby ensuring the trajectory remains consistent over time.
6. **Repeat from step 1**: The tracking process is sustained as long as the number of extracted line-segment points remains sufficient. Should the number of features diminish, a re-extraction process is initiated, preserving the previously robust tracked features and repeating the process from step 1.

**Figure 4:**
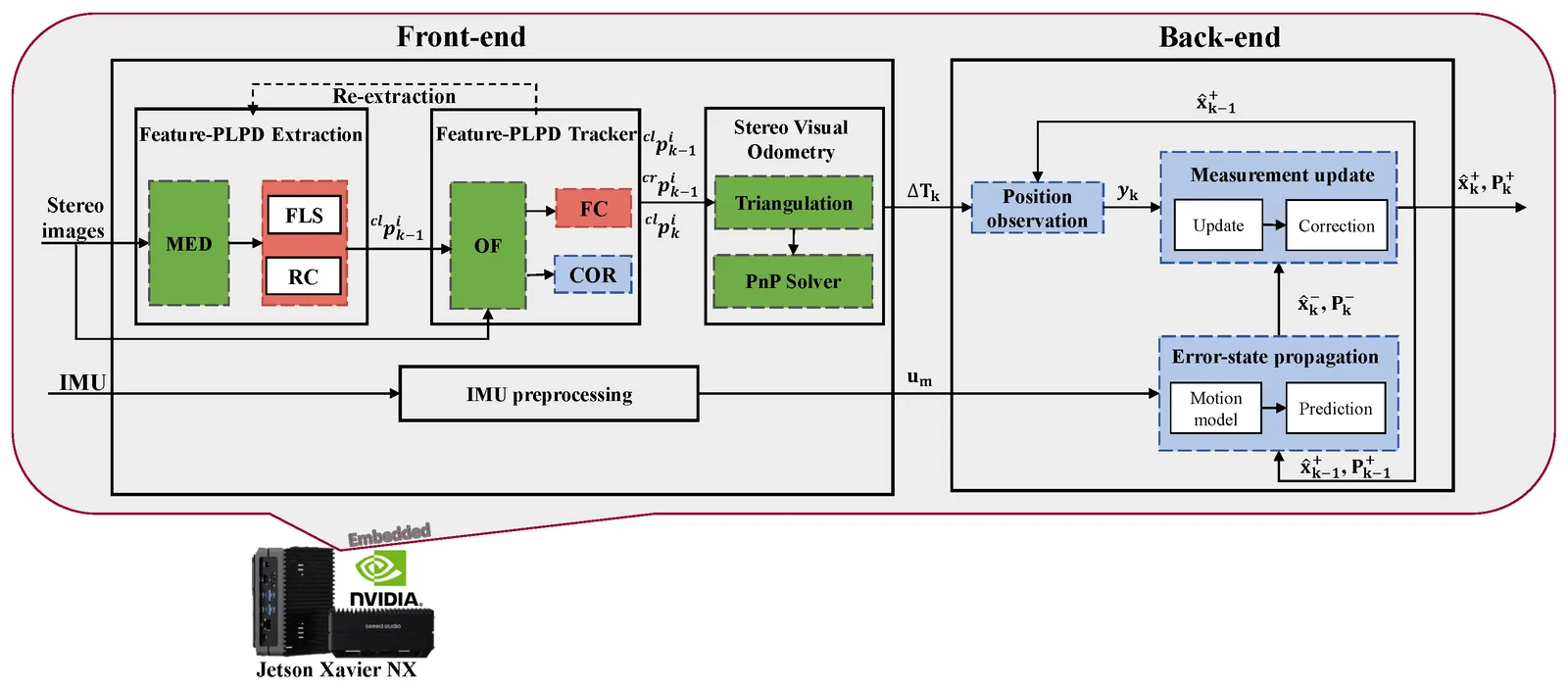
Feature-PLPD stereo VIO flowchart based on GPU-aware software design.

To deploy the proposed Feature-PLPD stereo VIO system on the embedded NVIDIA Jetson Xavier NX, the processing pipeline is organized as illustrated in Figure 4. The front-end exploits the heterogeneous architecture of the platform by leveraging CPU/GPU acceleration through CUDA for the blocks highlighted with green dashed lines, while multi-threading using OpenMP is applied to the blocks indicated with red dashed lines in order to parallelize computationally intensive tasks. In contrast, the back-end thread implements the ESEKF-based state estimator entirely on the CPU using C++, which is preferred for its efficiency in terms of resource usage and time savings.

The choice of stereo vision instead of a monocular configuration is primarily motivated by the targeted platform resource usage and the need to overcome scale ambiguity, which is an inherent limitation in monocular VIO.

### 3.4. Low-Cost Embedded Feature-PLPD Monocular VIO with Depth Scale Correction on FPGA-Based Heterogeneous Architecture

**Figure 5:**
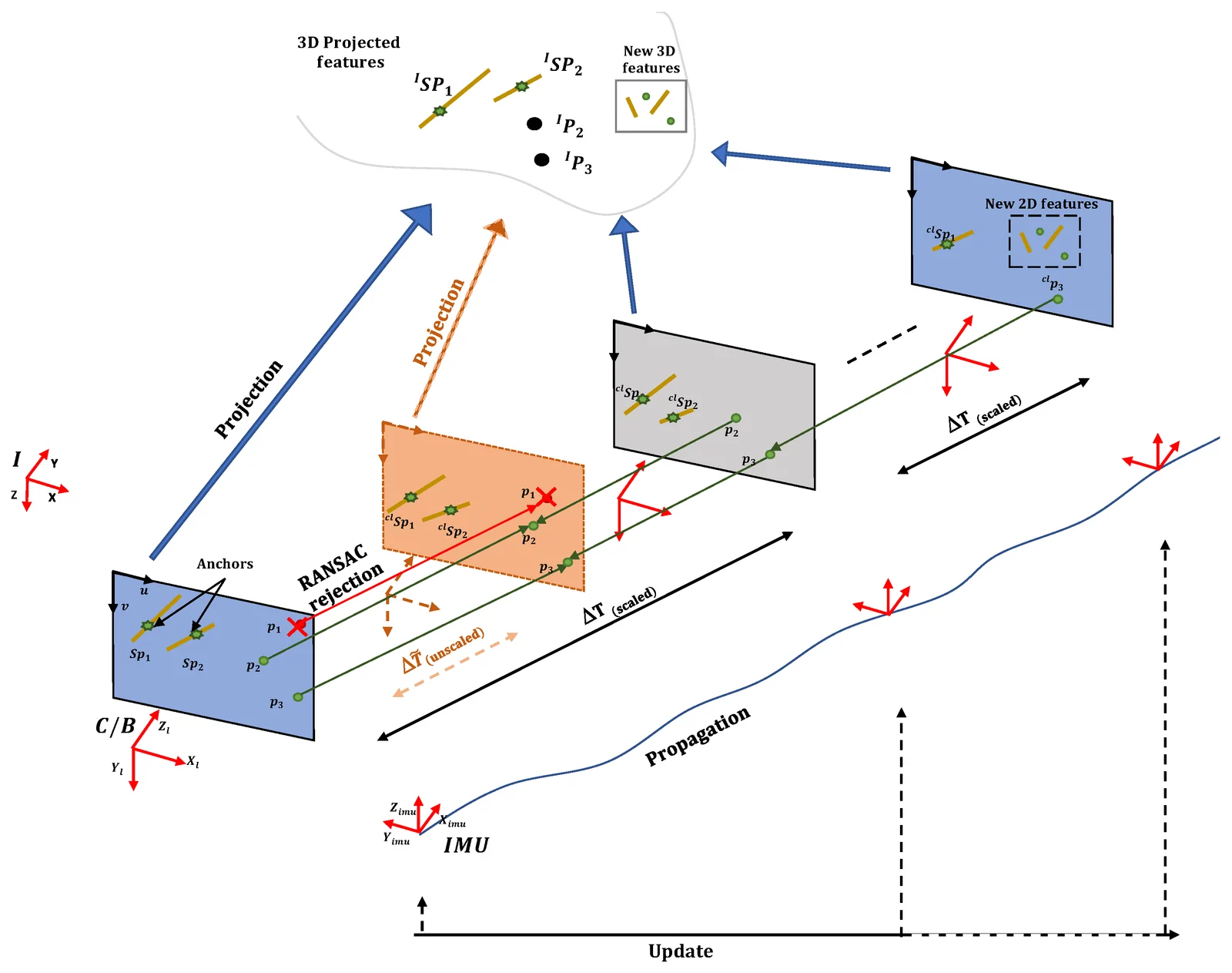
Feature-PLPD RGB-D-assisted monocular VIO process illustration with depth scale correction in orange parts. Blue frames indicate the feature extraction phase, while gray frames denote the feature tracking phase. Green and red lines indicate retained and rejected tracked features, respectively, orange arrows indicate the depth-assisted projection used for scale correction, and dashed lines indicate the continuity of the process across successive steps.

Building upon our previous end-to-end embedded Feature-PLPD stereo VIO solution, this section focuses on ensuring the portability of our proposed Feature-PLPD tracking for low-cost embedded architectures by reducing computation and resource usage. We propose a monocular version with depth scale correction (instead of triangulation through stereo vision), emphasizing the use of the proposed feature line tracker while refining the corner selection and rejection process. Figure 5 illustrates the proposed RGB-D-assisted monocular version, which follows these steps, assuming the body frame is the camera frame:

1. **Extraction**: We implement a stringent extraction and selection process for a set of features (corners and line-segment points) using the proposed Feature-PLPD from the previous camera frame. This solution primarily focuses on line-segment points rather than corners, as we have already fitted a model for controlling the tracking of these points in the next step.
2. **Tracking**: The forward feature tracking step is crucial for system accuracy. It is important to note that tracking line segments using the proposed OF-based flow correction method effectively maintains the alignment of line-segment points. In contrast, corner tracking is discouraged since corners do not have a fitted model as segments do and are only used in the absence of segments in certain scenes. Therefore, we adopt a strict corner outlier rejection process based on RANSAC, utilizing the geometric information obtained in the next step.
3. **Essential matrix computation**: We normalize the tracked features to compute the essential matrix between two frames.
4. **SVD**: We decompose the essential matrix using SVD to recover the unscaled estimated relative pose, denoted as $\Delta T_{unscaled}$ (refer to the orange parts in Figure 5).
5. **Depth scale correction and update**: To address the scale ambiguity inherent in monocular VO, we apply metric scale correction using depth measurements from an RGB-D sensor, similarly to the approaches described in [25,51]. The scale estimation relies on the feature correspondences retained after the tracking and geometric outlier-rejection steps described above. Before computing the scale, invalid or unreliable depth measurements are discarded, with the upper depth bound limited to 3 m, consistently with the reliable operating range considered for the RealSense D435i [52]. The scale factor is then estimated from the remaining valid correspondences by comparing their 3D norms, following [53,54]:
  (13) $$s=\frac{1}{N}\sum_{i=1}^{N}\frac{∥P_{real,i}∥}{∥P_{monoVO,i}∥}.$$
  Here, *N* denotes the number of valid correspondences retained after the tracking, geometric outlier-rejection, and depth-validity checks, $P_{real,i}=Z_{k,i}K^{-1}p_{k,i}$ is the measured 3D position of feature $p_{k,i}$ in the current frame using its depth $Z_{k,i}$, and $P_{monoVO,i}=\Delta rot\left(Z_{k-1,i}K^{-1}p_{k-1,i}\right)+\Delta tr$ is the corresponding 3D position predicted from the previous frame using the unscaled monocular motion $\Delta T_{unscaled}=\left[\Delta rot|\Delta tr\right]$.
  To reduce frame-to-frame fluctuations, the estimated scale factor is temporally smoothed using the four previous scale estimates through median filtering and interpolation. The resulting scale factor is then applied multiplicatively to the unscaled translation, while the relative rotation remains unchanged:
  (14) $$\Delta t_{scaled}=s\;\Delta t,\;\Delta T_{k}=\left[\begin{array}{cc} \Delta R & \Delta t_{scaled}\\ 0^{T} & 1 \end{array}\right].$$
  Although metric depth is available, it is used here only to recover the translation scale, thereby preserving the original Feature-PLPD motion estimation based on 2D correspondences and limiting the dependence on depth measurements. Accordingly, this configuration is referred to as RGB-D-assisted monocular VIO rather than conventional monocular VIO, since depth provides an external metric-scale reference instead of relying exclusively on visual–inertial information for scale recovery. This assistance requires additional depth access and validity checks, while preserving the underlying monocular Feature-PLPD motion-estimation structure. The reported results should therefore be interpreted within this RGB-D-assisted setting when compared with conventional monocular VIO systems. In practice, this formulation also provided more stable relative-rotation estimates than direct RGB-D PnP, which exhibited orientation ambiguities for some feature configurations.
  The resulting scaled relative pose $\Delta T_{k}$ is then used to compute the current visual measurement $y_{k}$ according to Equation (10). Although the scale factor could in principle be estimated from inertial measurements, the present implementation favors depth-based visual information. Depth observations provide a direct metric reference for the tracked features in the controlled indoor environment, whereas scale recovery from the low-rate IMU measurements would be more sensitive to noise, bias, and accumulated drift.
6. **IMU propagation**: Concurrently, the IMU data is propagated through the ESEKF-based observer in the back-end across various frames to update the state vector, which includes position, orientation, and velocity.
7. **Repeat from step 1**: The tracking process continues as long as there are sufficient extracted line-segment points. If the number of features diminishes, a re-extraction process is initiated, preserving previously robust tracked features and restarting from step 1.

**Figure 6:**
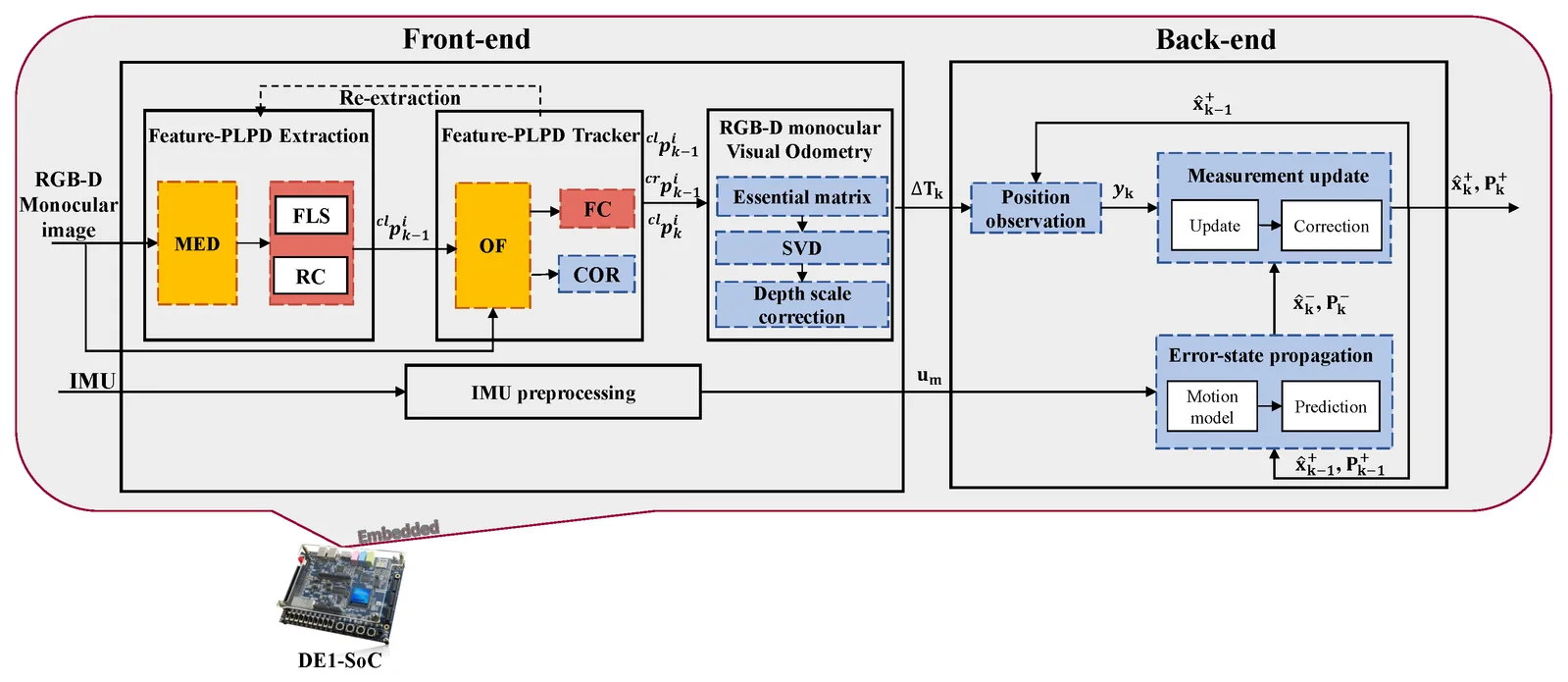
Feature-PLPD RGB-D-assisted monocular VIO flowchart based on FPGA hardware–software co-design.

To summarize, the proposed Feature-PLPD RGB-D-assisted monocular VIO system leverages the advantages of line-segment points rather than relying solely on corner features, while incorporating depth observations from low-resolution RGB-D images to resolve the scale ambiguity inherent to monocular VO. This design removes the computational overhead associated with stereo matching while maintaining reliable depth information, making the approach particularly suitable for deployment on resource-constrained platforms such as the targeted low-cost DE1-SoC FPGA-based heterogeneous architecture. To further reduce computational complexity, an amended OF algorithm supported by OpenCL is adopted, where pyramidal iterations are omitted and a rigorous feature selection and rejection strategy is applied to maintain robust tracking with limited resources. As illustrated in Figure 6, the system follows an FPGA hardware/software co-design paradigm, where the front-end thread exploits OpenMP acceleration on the ARM processor and OpenCL-based acceleration on the FPGA (represented by dashed red and yellow blocks, respectively).

The back-end relies on the ESEKF-based state estimator previously described, enabling efficient fusion of visual depth observations with inertial measurements while preserving real-time performance. This architecture effectively distributes the workload between the ARM processor and FPGA fabric, maximizing parallelism and pipelining while ensuring that the complete VIO pipeline remains compatible with the constraints of low-cost embedded systems.

## 4. Experimental Results

This section presents the quantitative and qualitative evaluation of the proposed Feature-PLPD VIO solutions. Two embedded implementations are considered: a stereo-based system on the Jetson Xavier NX and an RGB-D-assisted monocular system on the DE1-SoC. The analysis focuses on the three key aspects targeted in this work: trajectory accuracy, runtime performance, and energy consumption. The Jetson Xavier NX implementation is evaluated both offline using a stereo inertial benchmark dataset and on-the-fly through real-time deployment on a mobile robot. By contrast, the DE1-SoC implementation is assessed offline using pre-recorded sensor data, although the complete VIO pipeline is executed on the physical DE1-SoC platform, with the accelerated visual-processing kernels mapped onto the FPGA fabric and the remaining processing stages executed on the ARM processor. An on-the-fly deployment was not performed in the present study because the DE1-SoC was not yet integrated with the RealSense sensor and the mobile robotic platform. Such integration requires a RealSense-compatible acquisition layer on the HPS to acquire the RGB-D and inertial streams while preserving their timestamp-based temporal association, together with the required sensor-interface software support and power-supply integration. Once acquired, the sensor data would follow the same hardware/software processing partition evaluated offline, with the accelerated visual-processing kernels executed on the FPGA fabric and the remaining VO/VIO stages on the ARM processor. The evaluation is therefore restricted to dataset-based trajectory estimation together with runtime and FPGA-side energy analysis. The measured real-time processing capability nevertheless provides the computational basis for future on-the-fly integration, which constitutes a next step of this work.

### 4.1. Experimental Setup

#### 4.1.1. Platform Description

The first platform is the embedded heterogeneous reComputer J2021, based on the NVIDIA Jetson Xavier NX module (NVIDIA Corporation, Santa Clara, CA, USA). This board is a high-performance, low-power embedded platform that integrates a 6-core NVIDIA Carmel ARM v8.2 64-bit CPU and a Volta-based GPU with 384 CUDA cores and 48 Tensor cores, together with 8 GB of LPDDR4x memory. This platform was selected because it offers sufficient computational power and memory bandwidth to support real-time stereo visual–inertial processing on an embedded system.

The second platform is the DE1-SoC board, which represents an embedded FPGA-based heterogeneous architecture based on the Cyclone V SoC FPGA (Intel Corporation, Santa Clara, CA, USA). It combines a dual-core ARM Cortex-A9 processor (Arm Limited, Cambridge, UK) running at 800 MHz with FPGA programmable logic resources, including approximately 85K logic elements, 87 DSP blocks, and 4.45 Mb of embedded memory, as well as 1 GB DDR3 SDRAM. The DE1-SoC was selected to investigate the feasibility of deploying the proposed VIO pipeline under strict cost, resource, and power constraints in monocular processing mode.

#### 4.1.2. Datasets

**Figure 7:**
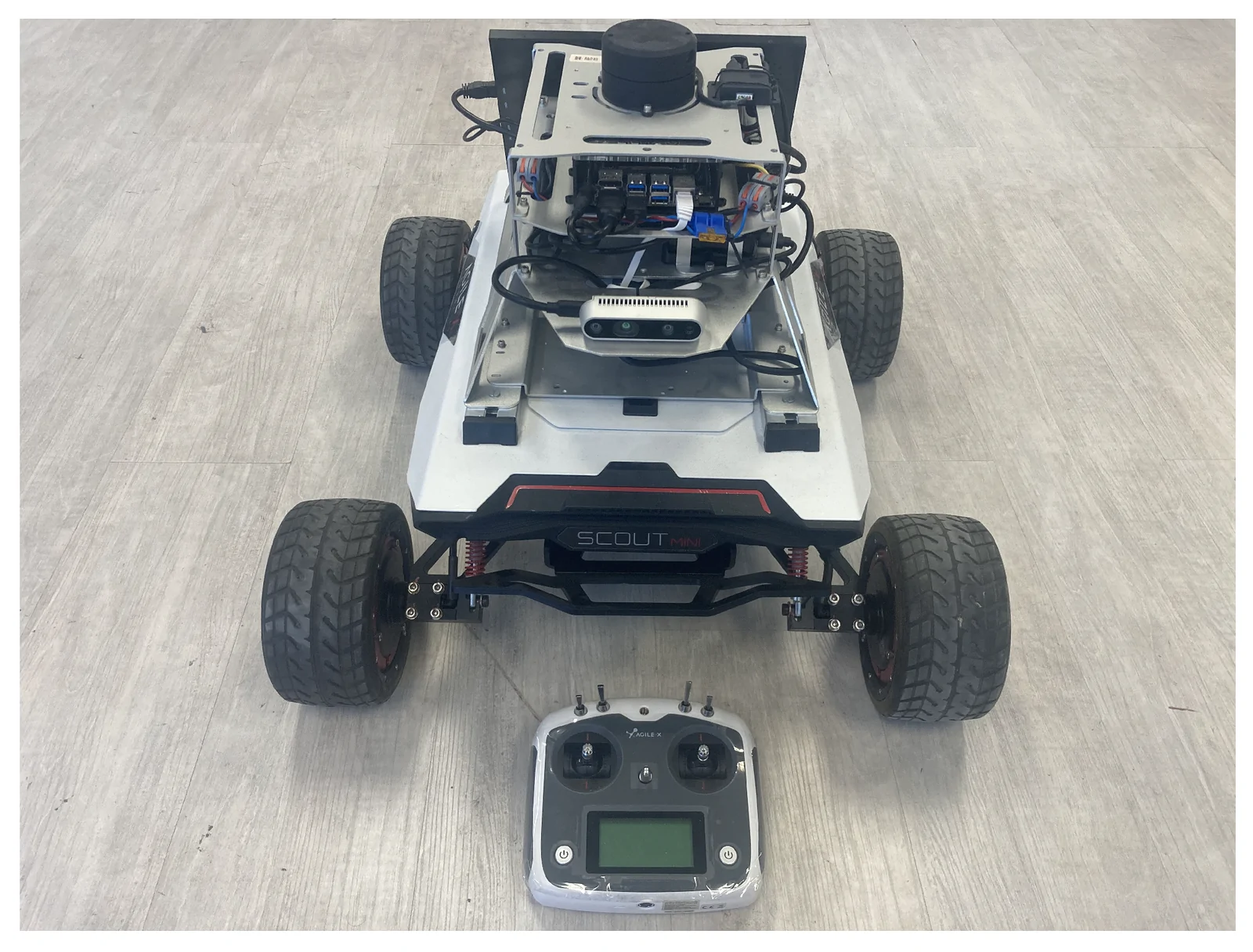
Experimental platform: Scout Mini equipped with a RealSense D435i camera and an embedded NVIDIA Jetson Xavier NX.

For the experimental evaluation ensuring consistent sensor fusion, visual and inertial data are temporally aligned at the image acquisition rate. Both VICON datasets were acquired using the RealSense D435i at 10 Hz due to the constraints of the indoor motion-capture environment, particularly to mitigate the periodic illumination variations induced by the infrared cameras of the VICON system, rather than due to the computational capability of the proposed pipeline. The IMU samples are hardware-timestamped using the depth-sensor clock, providing a common temporal reference between the inertial and depth streams. No additional camera–IMU extrinsic calibration is performed, as the rigid transformation between the IMU and the depth sensor is precalibrated in the device and internally applied by the RealSense SDK, such that the resulting inertial measurements share the depth-camera coordinate system (*X*-right, *Y*-down, and *Z*-forward). As shown in Section 4.2 and Section 4.3, the embedded implementations sustain approximately 20 fps. The datasets used for validation are described below:

1. **Outdoor KITTI stereo inertial dataset**: This dataset provides stereo image sequences with a resolution of 1241×376 and IMU measurements extracted from the corresponding raw KITTI data. For each image timestamp, the temporally nearest IMU measurement is selected from the raw sequence, without averaging or interpolation. The selected IMU measurements are transformed into the camera coordinate frame using the corresponding extrinsic calibration provided with the KITTI dataset, ensuring consistency with the visual measurements used by the VIO framework. This nearest-neighbor association discards the intermediate high-rate inertial measurements and may therefore reduce the representation of fast motion dynamics; this limitation is consistent with the camera-rate fusion trade-off discussed in Section 3.2. A controlled comparison with full-rate IMU propagation or pre-integration requires a temporally consistent high-rate inertial stream aligned with the evaluated odometry sequences. For the considered KITTI sequences, reconstructing such a stream from the corresponding raw inertial data is subject to data-continuity and synchronization constraints, which prevent the effect of the propagation rate from being isolated reliably. Consequently, the present evaluation is restricted to the synchronized camera-rate configuration, and no equivalence with full-rate inertial propagation or pre-integration is claimed.
  The OXTS data are processed using the official KITTI development tools and calibration parameters. Although the inertial measurements used by the VIO estimator and the ground-truth trajectory originate from the same OXTS unit, only the inertial measurements are provided as inputs to the proposed VIO framework, while the GPS-derived trajectory is used exclusively as ground truth for evaluation and is not involved in the state estimation. Ground truth is available for both translation and rotation, enabling a complete evaluation of the estimated trajectory in outdoor conditions. It should be noted that the OXTS RT3003 used in KITTI is a higher-grade navigation system and is therefore not representative of the low-cost IMUs primarily targeted in this work. This limitation is complemented by the indoor experiments using the integrated IMU of the RealSense D435i, which provide an evaluation with a more representative low-cost sensing configuration. The rectified sequences and their corresponding raw sequences are:
  - Sequence 00: 2011_10_03_drive_0027;
  - Sequence 05: 2011_09_30_drive_0018;
  - Sequence 06: 2011_09_30_drive_0020;
  - Sequence 10: 2011_09_30_drive_0034.
  These sequences were selected to limit the confounding influence of highly dynamic scenes and to provide consistent visual, inertial, and ground-truth data for evaluating the effect of visual–inertial fusion under identical conditions. This controlled selection enables the analysis to focus on the proposed embedded VO/VIO framework and its accuracy–runtime–energy trade-offs, rather than on dynamic-object handling, which is beyond the scope of this work. Consequently, the present KITTI evaluation should be interpreted as a controlled assessment of the proposed embedded VIO architecture rather than a comprehensive evaluation under highly dynamic or visually challenging conditions.
2. **Indoor VICON stereo inertial dataset**: Captured using the RealSense D435i (Intel Corporation, Santa Clara, CA, USA) in stereo mode and mounted on the Scout Mini R&D Kit, the dataset provides synchronized stereo images and inertial measurements for real-time robotic evaluation. Ground-truth translational motion is captured with high precision using the VICON system. The Scout Mini platform, illustrated in Figure 7, integrates an NVIDIA Jetson Xavier NX and enables on-the-fly processing of stereo image sequences at a resolution of 848×480.
3. **Indoor VICON RGB-D dataset**: Also captured using the RealSense D435i, this dataset uses the RGB-D configuration at a resolution of 424×240. It is used for the offline evaluation of the RGB-D-assisted monocular VIO implementation. Ground truth is obtained from the VICON system, with only translational data available.

#### 4.1.3. Evaluation Metrics

The evaluation metrics reported in Table 4 are used to assess the accuracy, runtime performance, and energy efficiency of the proposed VIO systems. For the outdoor evaluation using the KITTI dataset, both the translation (tr) and rotation (rot) components of the Absolute Trajectory Error (ATE) are reported. In contrast, for the indoor experiments conducted with the VICON dataset, only the translation component of ATE is considered, as rotational ground-truth data were not available in the recorded sequences. In addition, the Relative Pose Error (RPE), which measures the local drift of the estimated trajectory, is reported only for the low-cost RGB-D-assisted monocular VIO solution in order to further analyze its short-term motion estimation accuracy. The translational RPE is computed between consecutive poses, corresponding to a one-frame interval (0.1 s at the 10 Hz acquisition rate).

For the GPU-based runtime and power evaluations, each experiment was executed eleven times. The first execution was treated as a warm-up run and excluded from the reported results to avoid the transient overhead associated with GPU initialization and the initial kernel execution. The reported runtime, throughput, and Jetson power values were therefore obtained by averaging the ten subsequent executions under stable operating conditions.

Regarding energy evaluation, power consumption on the Jetson platforms (Jetson Xavier NX and Jetson Nano) is measured using the Monolithe energy measurement platform [55] developed at LIP6 laboratory, which provides real-time measurements of current, voltage, and power consumption. In contrast, for the DE1-SoC FPGA platform, direct hardware measurements were not available. Therefore, power consumption is estimated using the Intel Quartus PowerPlay Power Analyzer 14.0 in post-fit mode, which evaluates the dynamic and static power dissipation of the synthesized FPGA design based on device resource utilization, switching activity estimation, and timing information. Consequently, this estimate is restricted to the FPGA device and does not include the HPS or external DDR3 memory. Power associated with FPGA I/O activity is accounted for by the PowerPlay model, whereas the system-level energy associated with data transfers through external components is not captured.

Although the Jetson and DE1-SoC energy values are obtained using different measurement methodologies and system boundaries, they are used here only to provide an accelerator-level architectural comparison rather than a direct physical power comparison. In particular, the analysis focuses on energy per processed frame, which normalizes power consumption by the achieved throughput and provides a consistent metric for comparing heterogeneous embedded implementations.

### 4.2. End-to-End Embedded Feature-PLPD Stereo VIO

This subsection reports the experimental results obtained for the embedded stereo Feature-PLPD VO/VIO implementation on the Jetson Xavier NX, including runtime performance, trajectory accuracy, and energy consumption.

#### 4.2.1. Runtime Analysis

To better understand the computational efficiency of the proposed system, Table 5 summarizes the execution times, highlighting the benefits of the GPU-aware software design through a hybrid use of parallelization strategies. The most computationally demanding blocks, MED and OF, are offloaded to the GPU using CUDA 10.2, enabling massive parallel execution. Meanwhile, blocks FLS, CR, and FC are parallelized on the ARM via OpenMP, optimizing multi-threaded performance. Notably, the ESEKF prediction and update steps exhibit negligible latency, ensuring minimal delay in the back-end processing. This efficient architecture results in a throughput of 22 fps on KITTI and 21 fps on VICON, both comfortably exceeding the 10 fps acquisition rate of synchronized image and IMU data, and remaining within the acceptable limit for real-time embedded operation (tested up to 20 fps acquisition).

#### 4.2.2. Performance Evaluation

To assess the localization performance of the proposed Feature-PLPD stereo VIO system, the evaluation is conducted in two stages. First, a system-level incremental evaluation on KITTI sequence 00 analyzes the successive developments leading to the final system. All configurations are evaluated on the same sequence and Jetson Xavier NX platform under identical experimental conditions. Second, the final VO and VIO configurations are evaluated across multiple outdoor KITTI sequences and in the indoor VICON environment to assess the effect of inertial fusion under different operating conditions.

Table 6 traces the successive developments leading to the final VIO system. The original Feature-PLPD VO jointly detects and propagates corner and line-segment features to provide a hardware-aware visual front-end [21]. The enhanced line-only configuration subsequently exploits the linear structure of line segments through a dedicated optical-flow tracking strategy [22], reducing the translation ATE from 9.70 m to 7.87 m and increasing the throughput to 50 fps.

The final visual front-end combines the complementary processing strategies of these two developments: corners are propagated using the original Feature-PLPD strategy, while line-segment points benefit from the enhanced tracker. This combination achieves a translation ATE of 8.90 m, which is higher than the 7.87 m obtained with the enhanced line-only configuration on KITTI 00. This isolated result may be related to the different persistence mechanisms of the two visual primitives. The enhanced line tracker explicitly exploits the linear structure of segments and the common gradient properties of their constituent points to control and correct tracked line-segment points, allowing reliable correspondences to be maintained over longer portions of the sequence. This mechanism is particularly effective on KITTI 00, which contains abundant persistent linear structures. In contrast, corner features do not benefit in the present front-end from an additional structural model for correcting their tracked positions. Their inclusion may therefore affect the persistence and re-extraction behavior of the combined feature set. However, feature-level persistence statistics were not recorded in the present evaluation; therefore, this interpretation remains qualitative, and the lower ATE observed for the line-only configuration should not be interpreted as evidence of its general superiority. Nevertheless, relying exclusively on line features would make the system strongly dependent on the availability of persistent linear structures, which are particularly prevalent in man-made environments. Corners are therefore retained in the final point–line configuration as complementary visual information to improve the adaptability of the front-end to scenarios where such linear structures are sparse or unavailable.

The integration of the ESEKF-based inertial fusion then reduces the translation ATE from 8.90 m to 5.40 m and the rotation ATE from 0.16 rad to 0.13 rad, while maintaining a throughput of 22 fps. The system-level incremental evaluation therefore highlights the rationale behind the final system design: a more general point-and-line visual representation is retained for cross-scenario applicability, while resource-aware inertial fusion compensates for residual visual drift without compromising real-time execution. Overall, this evaluation traces the successive developments leading to the proposed VIO architecture and their respective contributions to the final localization performance.

Having analyzed the contribution of the successive developments, the final VO and VIO configurations are now evaluated across the selected outdoor and indoor scenarios.

**Figure 8:**
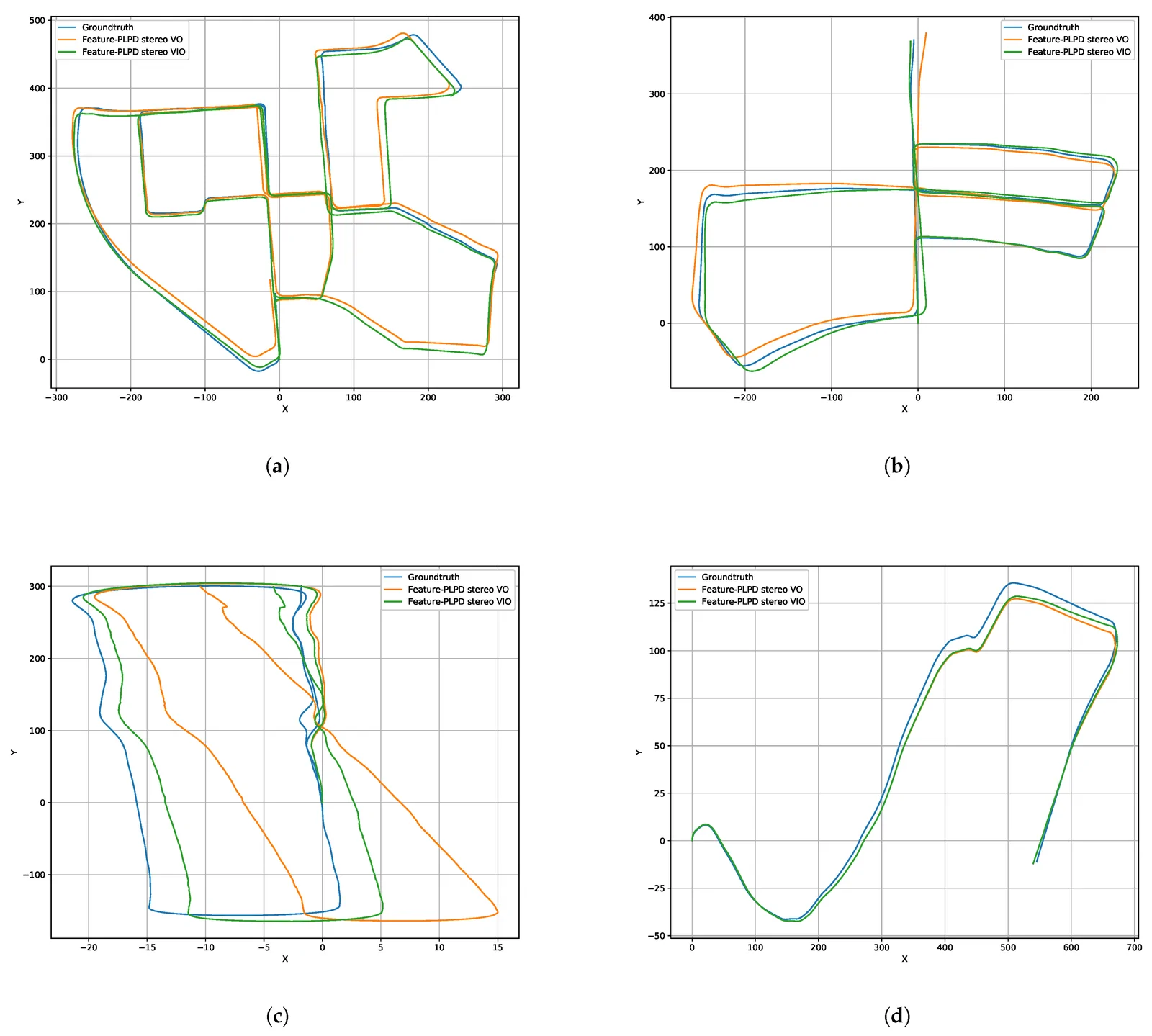
Feature-PLPD stereo VO/VIO trajectories on the outdoor KITTI dataset sequences: (**a**) 00, (**b**) 05, (**c**) 06, and (**d**) 10. The *X* and *Y* trajectory coordinates are expressed in meters.

Starting with qualitative assessment, the KITTI trajectories (see Figure 8) clearly show that the VIO version better tracks the ground truth, significantly reducing accumulated drift compared to the VO-only version. This improvement is especially noticeable during high-speed turns and in long trajectories where the VO system begins to deviate due to visual-only drift accumulation.

**Figure 9:**
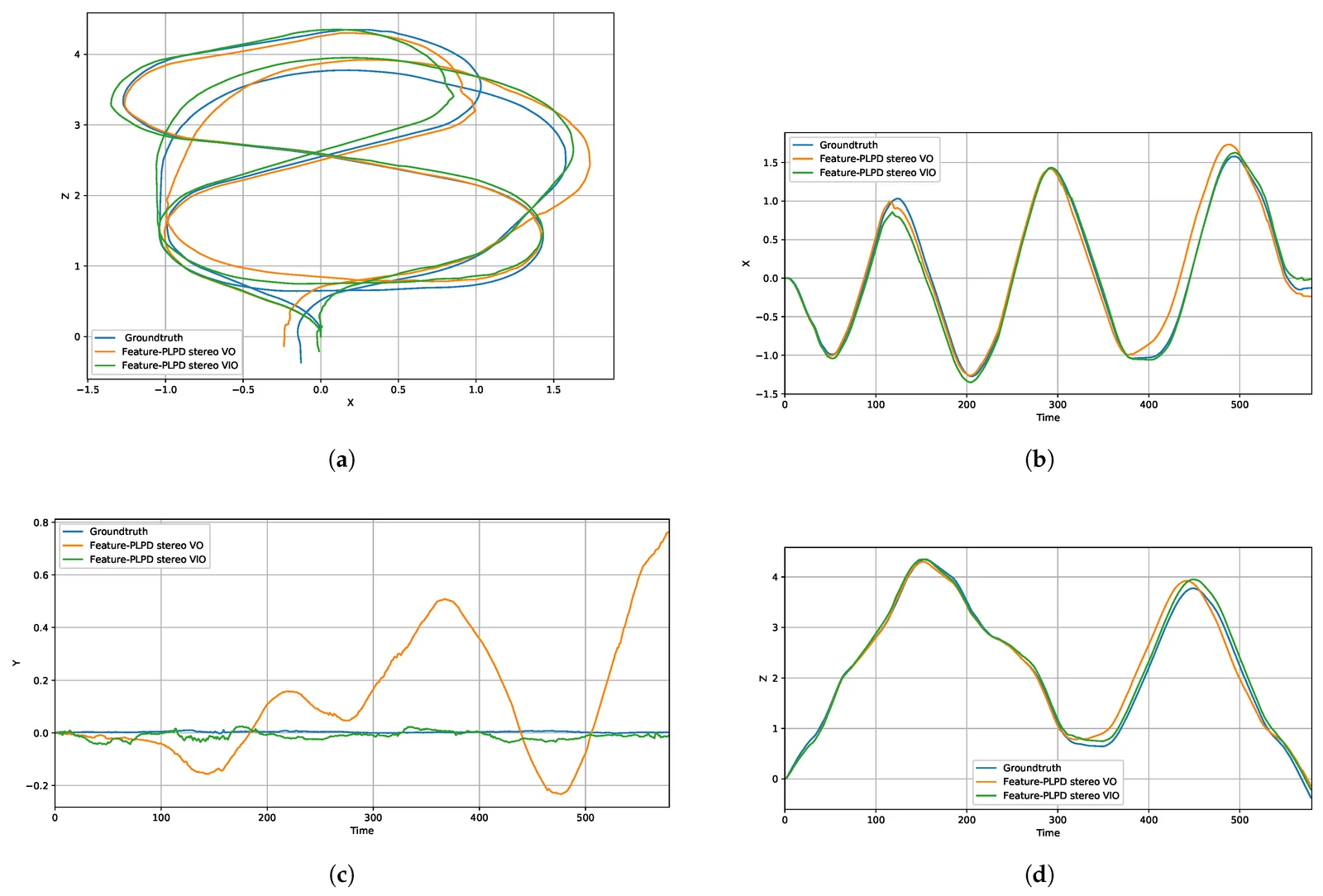
Feature-PLPD stereo VO/VIO results on the indoor stereo VICON dataset: (**a**) Trajectory in the *X*-*Z* plane, (**b**) *X*-axis evolution, (**c**) *Y*-axis evolution, and (**d**) *Z*-axis evolution. All position coordinates are expressed in meters.

In the VICON indoor environment, illustrated in Figure 9, the same trend is observed: the VIO version maintains close alignment with the ground truth along the X, Y, and Z axes, whereas the VO-only solution suffers from noticeable deviations after several frames, especially in Y-axis as shown in Figure 9c. These visual comparisons emphasize the benefit of inertial data fusion under controlled and constrained indoor movement.

To complement the qualitative analysis, Table 7 compares the final visual-only and visual–inertial configurations across all selected datasets. The VIO system consistently reduces both translational and rotational ATE on every KITTI sequence, although the magnitude of the improvement varies with the trajectory. Averaged over the four evaluated sequences, inertial fusion reduces the translation ATE by approximately 25% and the rotation ATE by approximately 18%.

The same trend is observed in the indoor VICON environment, where the translation ATE decreases from 1.35 m to 1.21 m, corresponding to an improvement of approximately 10%. For the 23.77 m ground-truth trajectory, these errors correspond to normalized ATE values of 5.68% and 5.09% for VO and VIO, respectively. Although rotational ground truth is unavailable for this experiment, the consistent reduction in translational error across both outdoor and indoor scenarios confirms that the ESEKF provides complementary motion information that limits visual-only drift under different operating conditions.

In addition to accuracy improvements, the embedded implementation maintains efficient runtime and energy characteristics. Given the negligible execution time of the ESEKF prediction and update steps reported in Table 5, the additional computational overhead introduced by inertial fusion is marginal compared with the visual front-end. The reported throughput and energy values therefore characterize the complete VIO implementation. The system achieves 22 fps on KITTI and 21 fps on VICON, exceeding the sensor acquisition rate and satisfying real-time requirements. The average power consumption at board level remains moderate at 8.1 W for outdoor experiments and 6.9 W for indoor experiments, corresponding to 368 mJ and 328 mJ per processed frame, respectively. These results demonstrate that the proposed GPU-aware software design achieves a favorable trade-off between accuracy, computational performance, and energy efficiency on the Jetson Xavier NX platform.

### 4.3. Low-Cost Embedded Feature-PLPD RGB-D-Assisted Monocular VIO

Following the evaluation of the Feature-PLPD stereo VO/VIO system implemented on the Jetson Xavier NX, this section shifts focus toward the monocular variant of the proposed solution. The goal is to explore how the same image pre-processing front-end and ESEKF-based back-end can be adapted for low-cost embedded platforms. In particular, we compare two distinct design paradigms: a GPU-aware software implementation on the Jetson Nano, and a FPGA-hardware/software co-design on the DE1-SoC FPGA. This comparison highlights the practical trade-offs of each design, where the Jetson Nano implementation leverages iterative OF computation across a three-level image pyramid and applies local thresholding in the MED block using CUDA, while the DE1-SoC implementation, constrained by limited resources, adopts a single-level pyramid for OF as provided by the Intel OpenCL and integrates the adaptive thresholding in MED block. Therefore, we targeted the 424 × 240 resolution and refined the FPGA-hardware MED co-design by reducing the Gaussian filter and structure tensor window size from 5 × 5 to 3 × 3. The resource report of this implementation on DE1-SoC FPGA is provided in Table 8.

#### 4.3.1. Runtime Analysis

Building on the architectural differences, the runtime evaluation, as shown in Table 9, reveals that the pipeline execution of MED and OF blocks on the FPGA using OpenCL 14.0 outperforms the parallel CUDA-based implementation on the Jetson Nano (NVIDIA Corporation, Santa Clara, CA, USA) (the OF pipeline uses only single pyramid level). However, the Jetson Nano compensates for this with a more powerful Quad-core ARM Cortex-A57, enabling faster execution of the other blocks compared to the DE1-SoC’s Dual-core Cortex-A9. The ESEKF-based back-end remains computationally negligible in both low-cost implementations. Overall, both systems achieve real-time performance, with 20.4 FPS on Jetson Nano and 19.7 FPS on DE1-SoC.

#### 4.3.2. Performance Evaluation

**Figure 10:**
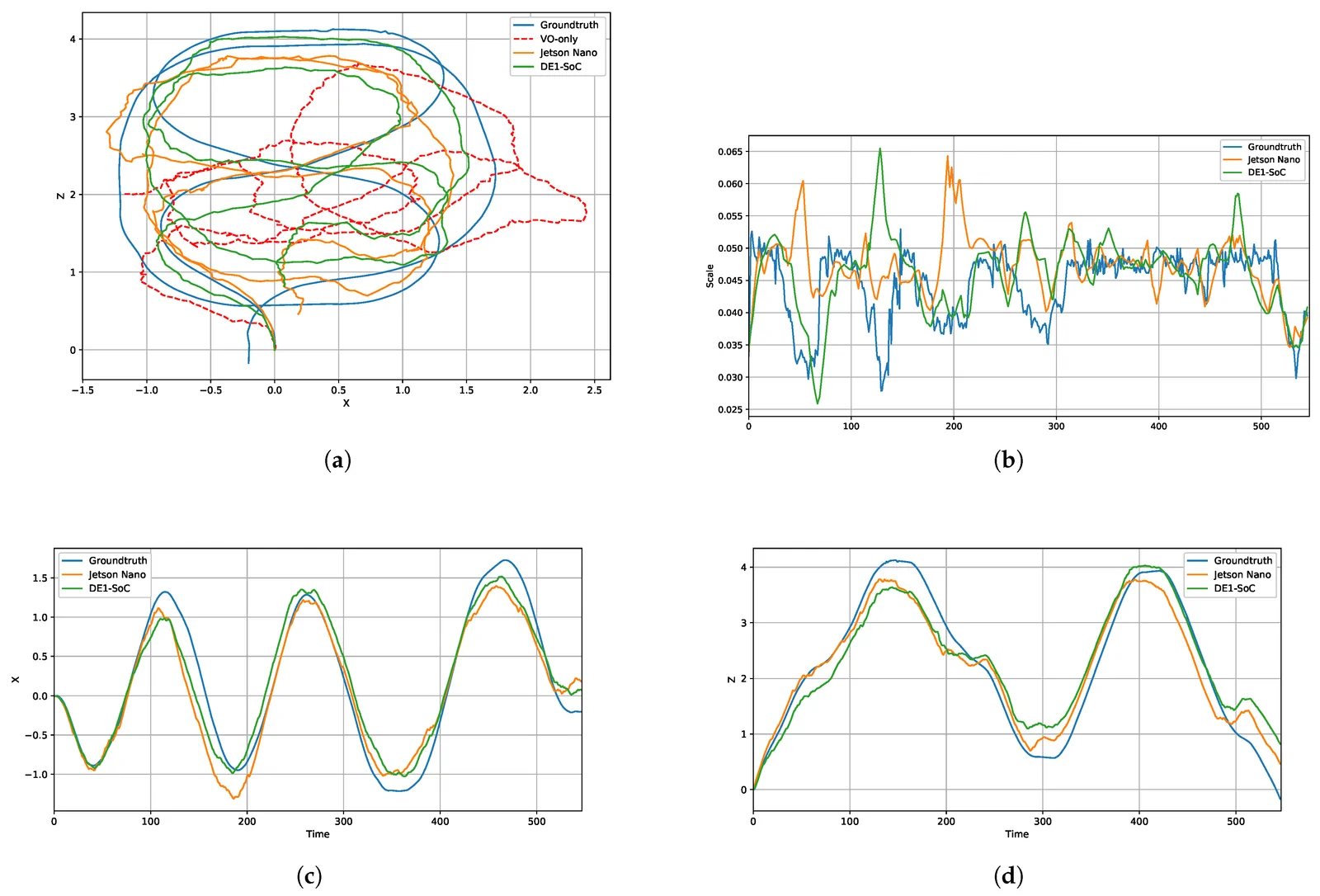
Benchmark of the Feature-PLPD RGB-D-assisted monocular VIO implementations on Jetson Nano and DE1-SoC using the indoor monocular VICON dataset: (**a**) Trajectory in the *X*-*Z* plane, (**b**) scale ambiguity, (**c**) *X*-axis evolution, and (**d**) *Z*-axis evolution. All position coordinates are expressed in meters.

For the qualitative assessment, the estimated trajectories and the evolution along the X and Z axes are illustrated in Figure 10a,c,d. Figure 10a additionally includes the VO-only trajectory to highlight the contribution of the proposed lightweight inertial fusion. The indoor RGB-D scenario is particularly challenging due to the combination of low-resolution images (424×240), monocular scale ambiguity (Figure 10b), illumination artifacts caused by the 100 Hz VICON motion-capture system, and the slow platform motion, which may result in weakly observable parallax and near-pure rotational motion. These conditions significantly affect visual-only pose estimation based on the essential matrix, as illustrated by the substantial drift of the VO-only trajectory. In contrast, both VIO implementations remain considerably closer to the VICON ground truth, demonstrating that the proposed ESEKF-based fusion effectively provides complementary motion information that improves robustness under challenging geometric conditions. These results show that, despite operating at the camera rate, the proposed inertial fusion provides a measurable improvement over the VO-only configuration under the considered experimental conditions.

Despite operating under resource-constrained embedded configurations, both VIO implementations successfully preserve the overall trajectory. The Jetson Nano implementation exhibits the smallest deviation from the ground truth, particularly during sharp turns and abrupt changes in motion direction, benefiting from its richer visual processing pipeline, including multi-scale tracking and adaptive thresholding. Although the DE1-SoC implementation operates under significantly tighter computational, memory, and energy constraints, it still preserves the overall trajectory shape with acceptable localization accuracy, demonstrating the feasibility of the proposed low-cost embedded VIO framework.

In contrast, the quantitative evaluation is performed using the ATE and the RPE computed from translation-only ground truth. As shown in Table 10, the Jetson Nano slightly outperforms the DE1-SoC in terms of accuracy, achieving an ATE of 1.24 m and an RPE of 0.047, compared to 1.26 m and 0.05 on the DE1-SoC. Both platforms are evaluated on the same 24.19 m VICON trajectory, corresponding to normalized ATE values of 5.13% for the Jetson Nano and 5.21% for the DE1-SoC. This difference is mainly attributed to the three-level pyramid OF and local thresholding executed on the GPU, which improve feature tracking under challenging visual conditions. In contrast, the DE1-SoC implementation relies on a single-level pyramid OF and global adaptive thresholding due to hardware resource constraints.

From an energy perspective, the total board-level power consumption of the Jetson Nano reaches 6.3 W (VDD_IN). This value is reported only as a system-level indicator and is not directly compared with the DE1-SoC power estimate. To provide an accelerator-level architectural comparison, energy efficiency is therefore analyzed by considering the measured VDD_CPU_GPU power domain on the Jetson Nano and the FPGA-side estimated power on the DE1-SoC, for which board-level measurement was unavailable. Since the most compute-intensive tasks are offloaded to these accelerators, the reported energy is computed from their power consumption and the cumulative execution time of the two corresponding GPU/FPGA kernels. Under this common functional scope, but considering the different measurement methodologies, the Jetson Nano requires 50.0 mJ, whereas the DE1-SoC is estimated at 15.8 mJ.

Overall, these results highlight the trade-off between the slightly higher localization accuracy of the GPU platform and the lower estimated accelerator-level energy of the FPGA-based implementation. Combined with a comparable real-time throughput of 20.4 fps and 19.7 fps, respectively, the results demonstrate the feasibility of deploying the proposed Feature-PLPD RGB-D-assisted monocular VIO pipeline on low-cost, resource-constrained FPGA-based embedded systems.

## 5. Conclusions

This paper presented a complete embedded Feature-PLPD VIO framework combining a hardware-aware point-and-line visual front-end with lightweight ESEKF-based fusion. Two complementary implementations were investigated: a performance-oriented stereo VIO on the Jetson Xavier NX and a frugality-oriented RGB-D-assisted monocular VIO on the DE1-SoC. The system-level incremental evaluation traces the successive developments leading to the final system and highlights the complementary roles of point and line processing and inertial assistance.

Evaluations in outdoor KITTI and indoor VICON environments confirm that the proposed lightweight fusion consistently limits visual-only drift. Averaged over the four KITTI sequences, the VIO configuration reduces the translation and rotation ATE by approximately 25% and 18%, respectively, while a 10% reduction in translation ATE is obtained over the indoor stereo VICON trajectory compared with the VO-only configuration. Relative to the corresponding trajectory lengths, the final VIO estimates achieve normalized ATE values of 5.09% for the stereo configuration and 5.21% for the RGB-D-assisted monocular configuration. Both embedded implementations sustain around 20 fps. The results further highlight the complementary objectives of the two architectures: the GPU-based solution favors higher accuracy, whereas the FPGA-based implementation exhibits lower estimated accelerator-level energy consumption under the considered measurement methodology. Overall, these findings show that practical embedded VIO should be addressed as a joint accuracy–runtime–energy design problem rather than solely as an accuracy optimization task.

Some limitations remain. The indoor experiments are affected by the illumination constraints of the VICON environment, while the RGB-D-assisted monocular configuration remains dependent on depth-based scale estimation and was evaluated offline on the DE1-SoC rather than through on-the-fly robotic deployment. In addition, the selected KITTI sequences limit the evaluation of the proposed framework under highly dynamic and visually challenging conditions. The experimental evaluation also does not include a controlled comparison between the adopted camera-rate fusion and full-rate IMU propagation or pre-integration; therefore, no equivalence between these inertial processing strategies is claimed. A direct quantitative comparison with existing complete VIO systems also remains outside the scope of the present experimental evaluation due to the heterogeneous experimental configurations considered across existing works. Future work will therefore extend the experimental validation to more dynamic scenarios and investigate this accuracy–resource trade-off using reliably synchronized high-rate inertial data, more robust scale estimation and synchronization, and the extension of the proposed algorithm architecture co-design toward tightly coupled embedded SLAM. In particular, persistent point and line landmarks will be investigated to provide stronger temporal and geometric constraints while preserving the resource-aware design objectives of the proposed approach.

## Figures and Tables

**Table 1:** Comparison of the previous Feature-PLPD developments and the present work.

Work	Algorithmic Contribution	Fusion	Embedded Implementation	Evaluation/Targeted Trade-Off
Feature-PLPD [21]	Hardware-aware joint corner and line-segment processing	N/A	ARM-GPU	VO accuracy–runtime trade-off
Enhanced line tracking [22]	Geometry-aware optical-flow correction for persistent line-segment tracking	N/A	ARM-GPU	Improved tracking accuracy and runtime
FPGA co-design [23]	Portability of the computationally intensive Feature-PLPD front-end	N/A	ARM-FPGA	Resource-efficient front-end implementation
Present work	Integration and extension toward complete point–line VIO	Resource-aware loosely coupled ESEKF	Stereo ARM-GPU VIO + RGB-D-assisted monocular ARM-FPGA VIO	Joint accuracy–runtime– energy trade-off under different embedded resource constraints

**Table 2:** State-of-the-art image pre-processing front-end performance in VO on Jetson Xavier NX using seq00 of KITTI dataset.

	VO			Corners				Segments			
Front-End	tr (m)	rot (rad)	T (fps)	Detect	F1 (%)	T (fps)	Match	Detect	A (pix)	T (fps)	Match
SOFT-VO [30]	12.56	0.17	**23**	GFT	39.2	82	OF	N/A	N/A	N/A	N/A
PL-SLAM [11]	12.02	0.22	2.2	ORB	13.6	44.2	BRIEF	LSD	20.8	5.5	LBD
PL-VIO [27]	32.65	**0.14**	2.4	FAST	31.2	**177**	OF	LSD	20.8	5.5	LBD
PLD-VINS [28]	18.04	0.19	18	GFT	39.2	82	OF	EDlines	30.1	15.6	OF
Ray-To-Ray [29]	33.15	0.21	12	ORB	13.6	44.2	BRIEF	EDlines	30.1	15.6	OF
Feature-PLPD [21]	**9.7**	0.17	22	PLPD	**44.2**	33	OF	PLPD	**50.3**	**31**	OF

*Note:* Bold values indicate the best result, while underlined values indicate the second-best result.

**Table 3:** System-level positioning of the proposed framework with respect to representative visual–inertial odometry systems.

Method	Fusion	Estimator	Visual Information	Inertial Handling	Evaluation Dataset/ Context	Computing Platform	Embedded/ on-Board Validation	Energy Evaluation
OKVIS [10]	Tightly coupled	Nonlinear optimization	Keypoints	IMU error terms/high-rate	EuRoC/own experiments	General-purpose CPU	Real-time system	Not reported
ROVIO [34]	Tightly coupled	EKF	Image patches	State propagation	Own indoor/UAV experiments	Single CPU core	On-board UAV, 20 Hz	Not reported
VINS- Mono [6]	Tightly coupled	Sliding-window optimization	Point features	IMU pre-integration	EuRoC + real-world experiments	Desktop CPU/mobile platform	MAV and mobile-device demonstrations	Not reported
PL-VIO [27]	Tightly coupled	Sliding-window optimization	Points and lines	IMU pre-integration	EuRoC + PennCOSYVIO	Intel Core i7-6700HQ, 2.60 GHz, 16 GB	Dataset evaluation	Not reported
PLD-VINS [28]	Tightly coupled	Sliding-window optimization	Points, lines, and depth	IMU pre-integration	OpenLORIS + real-world experiments	Xeon E5645/Intel NUC i7-8700K	Dataset + robot evaluation	Not reported
Proposed	Loosely coupled	ESEKF	Points and lines	Camera-rate prediction	KITTI + VICON	Jetson Xavier NX/Jetson Nano/DE1-SoC	GPU robotic deployment + FPGA hardware execution	Reported

**Table 4:** Evaluation metrics used in the experiments.

Category	Metrics	Unit
Accuracy	ATE: translation (tr), rotation (rot)	m, rad
	RPE: translation (tr)	m
Runtime	Average runtime	ms
	Average throughput	fps
Energy	Average power consumption	W
	Energy per frame	mJ

**Table 5:** The average runtime of the functional blocks of the proposed Feature-PLPD stereo VIO in millisecond (ms) on Jetson Xavier NX across outdoor KITTI and indoor VICON datasets.

			KITTI	VICON
Front-end	Extraction	MED	20.75	17.8
		FLS & CR	6.26	8.5
	Tracker	OF	14.4	12.8
		FC	4.1	3.86
		COR	0.03	0.04
	SVO	Triangulation	0.18	0.19
		PnP solver	4.63	3.82
Back-end	ESEKF	Prediction	0.021	0.023
		Update	0.027	0.022
Average throughput (fps)			22	21

**Table 6:** System-level incremental evaluation of the successive developments leading to the proposed Feature-PLPD VIO system on KITTI sequence 00 using the Jetson Xavier NX.

Configuration	ATE		FPS
	tr. (m)	rot. (rad)	
Feature-PLPD VO [21]	9.70	0.17	22
Enhanced line VO [22]	7.87	0.14	50
Combined Feature-PLPD VO	8.90	0.16	22
Proposed Feature-PLPD VIO	**5.40**	**0.13**	22

*Note:* Bold values indicate the best results.

**Table 7:** Evaluation of performance trade-offs for the proposed end-to-end Feature-PLPD stereo VIO on the Jetson Xavier NX.

		ATE				Energy		FPS
		VO		VIO				
Dataset/seq		tr	rot	tr	rot	W	mJ	
KITTI	00	8.9	0.16	**5.4**	**0.13**	8.1	368	22
	05	7.57	0.11	**6.1**	**0.09**			
	06	5.92	0.12	**4.68**	**0.1**			
	10	5.53	0.17	**4.8**	**0.14**			
VICON		1.35	N/A	**1.21**	N/A	6.9	328	21

*Note:* Bold values indicate the best results.

**Table 8:** Report of the DE1-SoC FPGA resource usage in (%).

Resource (%)	MED	OF	MED & OF
Logic gate	75	20	85
Logic register	33	14	42
Memory block	33	16	44
DSP block	71	24	82

**Table 9:** The average runtime of the functional blocks of the proposed Feature-PLPD RGB-D-assisted monocular VO/VIO in millisecond (ms) on Jetson Nano and DE1-SoC across indoor VICON dataset.

			Jetson Nano	DE1-SoC
Front-end	Extraction	MED	13	8
		FLS & CR	7.8	10.3
	Tracker	OF	10.8	2.5
		FC	7.1	15.3
		COR	0.01	0.01
	VO	Essential	8.4	11.8
		SVD & scale	2.05	2.87
Back-end	ESEKF	Prediction	0.031	0.05
		Update	0.029	0.04
Average throughput (fps)			**20.4**	19.7

*Note:* Bold value indicates the best result.

**Table 10:** Jetson Nano power corresponds to the measured VDD_CPU_GPU power domain, whereas DE1-SoC power corresponds to an FPGA-side post-fit PowerPlay estimation; energy values should therefore be interpreted as an accelerator-level architectural comparison rather than equivalent physical power measurements.

Platform	Translation Error		Energy		FPS
	ATE	RPE	W	mJ	
Jetson Nano	**1.24**	**0.047**	2.1	50	20.4
DE1-SoC	1.26	0.05	**1.5**	**15.8**	19.7

*Note:* Bold values indicate the best results.

## Data Availability

Data and code are available on request from the corresponding authors.

## Abbreviations

The following abbreviations are used in this manuscript:

A3	Algorithm Architecture Adequacy
ATE	Absolute Trajectory Error
BRIEF	Binary Robust Independent Elementary Features
COR	Corner Outlier Rejection
CR	Corner Refinement
CUDA	Compute Unified Device Architecture
DSO	Direct Sparse Odometry
EDLines	EDge Line-segment detector
ESEKF	Error-State Extended Kalman Filter
FAST	Features from Accelerated Segment Test
FC	Flow Correction
FLS	Find Line Segments
FPGA	Field Programmable Gate Array
FPS	Frame Per Second
GFT	GoodFeatureToTrack detector
GPU	Graphics Processing Unit
HOOFR	Hessian ORB - Overlapped FREAK
IMU	Inertial Measurement Unit
LBD	Line Band Descriptor
LiDAR	Light Detection and Ranging
LSD	Line-Segment Detector
MED	Multi-level Edge Detector
OF	Optical Flow
OpenCL	Open Computing Language
ORB	Oriented FAST and Rotated BRIEF Oriented FAST and Rotated BRIEF
PLPD	Point and Line Points Detection
RANSAC	Random Sample Consensus
RPE	Relative Pose Error
SLAM	Simultaneous Localization and Mapping
VIO	Visual–Inertial Odometry
VO	Visual Odometry
